# Biochemical Characterization and Genome Analysis of *Pseudomonas loganensis* sp. nov., a Novel Endophytic Bacterium

**DOI:** 10.1002/mbo3.70051

**Published:** 2025-08-13

**Authors:** Melisa Z. Karaman, Ahmet E. Yetiman, Jixun Zhan, Ozkan Fidan

**Affiliations:** ^1^ Department of Bioengineering, Faculty of Life and Natural Sciences Abdullah Gul University Kayseri Turkiye; ^2^ Department of Food Engineering Department, Faculty of Engineering Erciyes University Kayseri Turkiye; ^3^ Department of Biological Engineering, College of Engineering Utah State University Logan Utah USA

**Keywords:** endophytic bacteria, Pseudomonas, taxonomy, turnerbactin, zeaxanthin

## Abstract

*Pseudomonas* species are highly adaptable, thriving in diverse environments and exhibiting remarkable genetic and metabolic diversity. While some strains are pathogenic, others have significant ecological and industrial applications. Bioinformatics and biochemical analyses, including antibiotic sensitivity testing, revealed that *Pseudomonas loganensis* sp. nov. can tolerate NaCl concentrations up to 5% and pH ranges between 5 and 9. Antibiogram results corroborated genome data, demonstrating resistance to vancomycin, ampicillin, methicillin, oxacillin, and penicillin G. Phylogenetic analysis based on 16S rRNA, *rpoB*, *rpoD*, and *gyrB* genes, combined with average nucleotide identity (ANI) comparisons, confirmed *P. loganensis* sp. nov. as a novel species within the *Pseudomonas* genus. Genome analysis further revealed the presence of turnerbactin and carotenoid gene clusters. Turnerbactin, known to contribute to nitrogen fixation in plants, highlights the strain's potential as a biofertilizer. Additionally, the carotenoid gene cluster suggests potential applications in industrial carotenoid production. The discovery of a trehalose synthase (*treS*) gene indicates the capability for one‐step conversion of maltose into trehalose, underscoring its potential utility in trehalose production.

## Graphical abstract

*Pseudomonas loganensis* sp. nov., is a novel strain identified through genomic and biochemical analysis, exhibits salt and pH tolerance, and harbors biosynthetic gene clusters for turnerbactin, carotenoids, and trehalose, highlighting its potential in agriculture and industrial biotechnology.

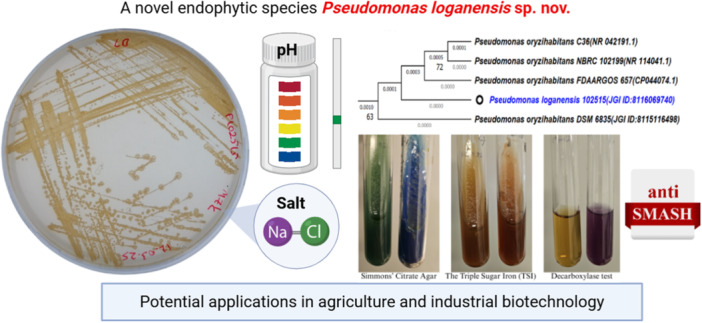

## 1 Introduction

The genus *Pseudomonas*, first described in 1894, consists of motile, rod‐shaped, aerobic, non‐spore‐forming, Gram‐negative bacteria belonging to the class *Gammaproteobacteria* (Palleroni 2005; Peix et al. 2018). More than 300 species have been characterized with validated taxonomic names (Rudra and Gupta 2023). *Pseudomonas* species are known to inhabit diverse ecological niches and environments, thus demonstrating remarkable genetic and metabolic diversity (Lalucat et al. 2020; Palleroni 2008). While some *Pseudomonas* species act as opportunistic pathogens of humans, animals, and plants, others hold significant industrial, economic, and ecological importance (Rudra and Gupta 2023). For instance, one of the human pathogens is *Pseudomonas aeruginosa* which causes infections in blood, lungs, urinary and gastrointestinal tracts, eye, ear, and other parts of the body. It might lead to the sepsis or chronic diseases (Qin et al. 2022; Stover et al. 2000; Wood et al. 2023). *P. anguilliseptica* infects wild and farmed fishes, leading to significant economic losses (Tawfeek et al. 2024). On the other hand, *P. aeruginosa* can also be used for the production of rhamnolipid, which is a biosurfactant and has potential applications in industry and environment such as surface coatings, additives for bioremediation, and biological control agent (Cerqueira dos Santos et al. 2024; Maier and Soberón‐Chávez 2000; Soberón‐Chávez et al. 2021). Moreover, the model *Pseudomonas* species, *Pseudomonas putida*, has been widely utilized as an important synthetic biology host and a metabolic engineering platform for the production of polymers, bulk chemicals, industrial enzymes, natural products, drug molecules, and high‐value specialties (Loeschcke and Thies 2015; Martínez‐García and de Lorenzo 2024; Rios et al. 2018; Wang et al. 2020; Weimer et al. 2020). Furthermore, *P. putida* has been genetically engineered to create a rapid, sensitive, and adaptable whole‐cell biosensor capable of detecting a broad range of diverse chemicals, offering significant benefits for environmental analysis (Hernández‐Sancho et al. 2024).

*Pseudomonas* species have been found in a diverse array of environments, including soil, water, sediments, air, plants, animals, fungi, algae, compost, and sources associated with humans and animals. Remarkably, some species have also been isolated from extreme habitats like Antarctica and the Atacama Desert (Peix et al. 2018). Furthermore, *Pseudomonas* species have also been isolated from understudied sources as endophytic and endolichenic bacteria (Ali et al. 2024; Devi et al. 2017; Fidan and Zhan 2019; Ibrahim et al. 2020; Lally et al. 2017; Wang et al. 2024). For instance, the lichen genus, *Peltigera*, was found to harbor *P. syringae* as an endolichenic bacterium, indicating its potential localization and survival beyond higher plants (Ramírez et al. 2023). Particularly, endophytic *Pseudomonas* species have been attracted attention of researchers since they were known to exhibit biocontrol efficacy and plant growth promoting activity (Devi et al. 2017; Ibrahim et al. 2020; Lally et al. 2017; Zeng et al. 2022). Additionally, they were reported to promote the biosynthesis of plant natural products (Wang et al. 2024). These features of endophytic *Pseudomonas* species propound the potential applications in agriculture. Some members of endophytic *Pseudomonas* species, such as *P. loganensis* sp. nov. (previously reported as *Pseudomonas* sp. 102515) were also reported as the efficient producers of certain secondary metabolites, indicating their potential applications for industrial production of value‐added products (Fidan and Zhan 2019).

In this study, the bioinformatics analysis and biochemical tests for the endophytic *P. loganensis* sp. nov. were conducted to further characterize the bacterium. For this purpose, various biochemical tests as well as antibiotic sensitivity analysis of the *P. loganensis* sp. nov. were investigated. In addition, the phylogenetic trees based on 16S rRNA, *rpoB*, *rpoD*, and *gyrB* genes and average nucleotide identification analysis revealed that *Psedumonas loganensis* sp. nov. is a putative novel *Psedumonas* species.

## 2 Materials and Methods

### 2.1 Bacterial Growth Conditions

*Pseudomonas loganensis* sp. nov. (also known as *Pseudomonas* sp. 102515) was previously isolated from the yew tree (*Taxus chinensis*) (Fidan and Zhan 2019). The endophytic *Pseudomonas loganensis* sp. nov. was revived from the −80°C glycerol stock (Fisher chemical). The growth conditions used in this study were at 28°C for 2 days in Luria‐Bertani (LB) agar and/or at 28°C for 1 day in LB broth (Condalab) at 220 rpm.

### 2.2 Biochemical Tests

The effects of different pH and NaCl concentrations on the growth of this bacterium were tested for pH and salinity tolerance. For the salinity tolerance test, the overnight‐grown inoculum culture was added to specifically prepared test tubes with 5 mL of LB and different NaCl (Fisher Chemical) amounts (0.1, 0.5, 1, 1.5, 2, 3, 4, 5, 6, 7, 8, and 9%) with 1% inoculum ratio. Similarly, for the pH tolerance test, the overnight‐grown inoculum culture was added to test tubes with 5 mL of LB with pH of 2, 3, 4, 5, 6, 7, 8, and 9 with 1% inoculum ratio. The pH of the medium in test tubes was adjusted by adding 37% HCl or 1 M NaOH (Fisher Chemical). Test tubes were incubated as triplicates at 28°C for 48 h. Absorbance values were measured at 600 nm with a spectrophotometer (Thermo Fisher, Genesys). *P. loganensis* sp. nov. was also characterized phenotypically using Odin, Biolog GEN III MicroPlate (Newark, DE 19702). The Phenotype MicroArray Plate 1 (PM 1) was used to test the growth of the bacterium against 95 different substrates including different carbon and nitrogen sources. Briefly, The *P. loganensis* sp. nov. was subcultured and struck for isolation on LB agar for 48 h at 28°C. The Biolog Gram‐negative PM protocol was followed by inoculating the organism in IF‐0a inoculating fluid to a density of 42% transmittance using a turbidimeter. Redox dye D was used to assess cellular metabolism. The cell suspension with redox dye was inoculated into the PM 1 plate at 100 μL per well. The plate was placed on Odin set to 28°C, and the kinetic data was collected every 20 min for 48 h.

Methyl Red and Voges‐Proskauer Test: Buffered peptone‐glucose broth, methyl red solution (Sigma‐Aldrich), and Voges‐Proskauer reagents were used. Voges‐proskauer reagents are Barritt's reagent A (5% (wt/vol) a‐naphthol (Merck) in absolute ethanol) and Barritt's reagent B (40% (wt/vol) NaOH (Merck Supelco) in deionized water). After the inoculation of the bacteria in a 5 mL MR‐VP broth and nearly 2 days of incubation, 2.5 mL of culture was transferred into a new tube and five drops of the methyl red reagent were added. Twelve drops of Barritt's reagent A and four drops of Barritt's reagent B were added into the remaining 2.5 mL of culture in the test tube. After gently shaking for 30 s to 1 min, test tubes were compared and analyzed within 1 h (McDevitt 2009).

Lysine Decarboxylase Broth Test: Commercial lysine decarboxylate broth (Oxoid) was used as tablets. One tablet was added to 5 mL of distilled water and sterilized by autoclaving. A single colony was transferred into the test tube with decarboxylase broth. After inoculation, the tube was incubated for 1 day and checked for color changes (Lal and Cheeptham 2015).

Indole Test: Tryptone peptone broth was prepared by using tryptone and NaCl in test tubes. Also, Kovác's reagent was prepared by 7.5 mL isoamyl alcohol (Tekkim), 0.5 g *p*‐dimethylaminobenzaldehyde (DMAB) and HCl. Test tubes were inoculated with 1% of fresh culture and incubated for ~2 days. Five drops of Kovác's reagent were directly added to the tube and tubes were checked for color change. Furthermore, an indole‐nitrite medium (BD BBL) was prepared with 0.5% agar in test tubes. A single colony was picked with a needle and then stabbed into agar. After ~2 days incubation, five drops of Kovác's reagent were added to the tube and tubes were checked for color change (MacWilliams 2012).

Triple Sugar Iron (TSI) Agar Test: After preparing TSI agar, a single colony was picked by using a straight inoculating needle and inoculated into prepared agar from deep to the upper part (Lehman 2005). Citrate Utilization Test: Simmons citrate medium (Difco BBL, USA) was prepared. After a single colony was picked by using a straight inoculating needle, the colony was inoculated into prepared agar from deep to the upper part (MacWilliams 2009). Motility Test: The overnight grown bacterial inoculum was submerged into the motility test medium (3 g/L beef extract, 10 g/L pancreatic digest of casein, 5 g/L sodium chloride, 4 g/L agar, 10 g/L dextrose, and 0.5 g/L 2,3,5‐triphenyltetrazolium chloride) using a straight inoculating needle and the tube was incubated at 28°C to observe for the color change (Shields and Cathcart 2011; Yetiman and Ortakci 2023).

### 2.3 Antibiotic Resistance Tests

*P. loganensis* sp. nov. was evaluated for antibiotic resistance using the disk diffusion method with triplicates (Bauer et al. 1966). The antibiotics tested included oxacillin (1 μg), methicillin (5 μg), kanamycin (30 μg), azithromycin (15 μg), penicillin G (10 μg), ampicillin (10 μg), tetracycline (30 μg), vancomycin (30 μg), amikacin (30 μg), and streptomycin (10 μg) (Oxoid). Antibiotic discs were placed on LB agar plates inoculated with 1% culture and then slightly frozen. After incubation at 28°C for 24 h, inhibition zones were measured with digital calipers (in mm), and results were categorized as susceptible (zone diamete ≥ 20 mm), intermediate (zone diameter between 15 and 19 mm), and resistant (zone diameter ≤ 14 mm) following the guidelines of the Clinical and Laboratory Standards Institute (CLSI. 2020 instructions (CLSI 2020).

### 2.4 Chemotaxonomic Analysis

The whole‐cell fatty acid methyl esters (FAME) analysis for *P. loganensis* sp. nov. was performed following the standard protocol (Sasser 1990). Briefly, after culturing the bacteria, approximately 40 mg of bacterial cells were harvested from the third quadrant of the streaked LB agar plate. Fatty acids were obtained through four stages: saponification, methylation, extraction, and washing. Approximately two‐thirds of the top phase from the washing step, which contained the clean extract, was transferred to a gas chromatography‐mass spectrometry (GC‐MS) sample vial for analysis. GC‐MS analysis was performed using a GCMS‐QP2010 Ultra (Shimadzu, Japan) with an Rtx‐5MS capillary column (30.0 m length × 0.25 mm diameter × 0.25 µm thickness). The column temperature was ramped from 40°C to 100°C for 1 min, with an injection temperature of 280°C in splitless mode. Helium was used as the carrier gas at a column flow rate of 2 mL/min, and the pressure was set to 112 kPa.

### 2.5 Bioinformatics Analysis

The whole genome of *P. loganensis* sp. nov. was recently announced with GenBank accession number of JAVHTY000000000.1 (Fidan 2024). This genome data was retrieved from NCBI and used in the bioinformatics analysis. Firstly, the 16S rRNA sequence of *P. loganensis* sp. nov. was analyzed using the NCBI‐BLAST core nucleotide and 16S ribosomal RNA databases, as well as the JGI‐IMG BLAST 16S rRNA database. Strains exhibiting above 95% similarity in 16S rRNA were selected for phylogenetic analysis. The 16S rRNA sequences used in this study were aligned with the MUSCLE algorithm (Edgar 2004) by using the UPGMA cluster method with 100 iterations in MEGA software v11.0.10. Later, phylogenetic tree reconstruction was carried out according to neighbor joining method using 1000 bootstrap replications and a maximum composite likelihood substitution model that included transitions and transversions via MEGA software v11.0.10 (Tamura et al. 2004, 2021). On the other hand, a multi‐locus sequence analysis (MLSA) phylogenetic tree was generated with the concatenated sequences of the 16S rRNA, *rpoB*, *rpoD*, and *gyrB* genes from previous *Pseudomonas* members under same alignment conditions (Frasson et al. 2017). Some of the sequences belonging to *rpoB*, *rpoD*, and *gyrB* genes that are not available from NCBI were retrieved from BV‐BRC v.3.39.10 (Olson et al. 2023). Evolutionary divergence of 16S rRNA and concatenated sequences were determined by pairwise distance computation by MEGA software v11.0.10 with similar parameters used in phylogenetic tree construction. An average nucleotide identity (ANI) analysis was fulfilled by JGI‐IMG/M amongst the *Pseudomonas* species (Chen et al. 2019). Moreover, a whole genome ANI comparison was conducted between *P. loganensis* sp. nov. and other closely related *Pseudomonas* species from aforementioned constructed phylogenetic trees, via the FastANI calculator v.1.33 with default parameters (https://gtdb.ecogenomic.org/tools/fastani) (Jain et al. 2018). The phylogenetic distribution of genes associated with cluster of orthologous groups (COG) functional categories of *P. loganensis* between *Actinomycetota* and *Pseudomonadota* exhibiting over 90% BLAST identity was determined via JGI/IMG‐M (Chen et al. 2017). BlastKOALA was used to automatically assign KEGG Orthology (KO) terms and to obtain KEGG mapping (Kanehisa et al. 2016). The genome of *P. loganensis* sp. nov. was screened against metagenome‐assembled genomes (MAGs) from JGI‐IMG/MER and Protologger databases (Chen et al. 2019; Hitch et al. 2021). A comprehensive description of *P. loganensis* sp. nov. was generated via Protologger Galaxy edition (https://protologger.bi.denbi.de). The percentage of conserved proteins (POCP) was compared with other *Pseudomonas* species utilizing Protologger. The digital DNA‐DNA hybridization (dDDH) analysis was carried out via DSMZ's web interface for the genome‐to‐genome distance calculator (Meier‐Kolthoff et al. 2022). *In silico* analysis of biosynthetic gene cluster were conducted using AntiSMASH 8.0 (Blin et al. 2025).

## 3 Results and Discussion

### 3.1 Origin of Bacterial Strain and Initial Identification

*Pseudomonas* species can exist in many environments, ranging from soil, water, and sediments to host species such as plants or animals. An endophytic bacterium was isolated from the leaves of *Taxus chinensis* collected from the campus of Utah State University in Logan, Utah and the initial identification through 16S ribosomal RNA (rRNA) amplification revealed that it was a *Pseudomonas* species and the closest relative to this endophytic isolate was *P. psychrotolerans* from BLASTn analysis in our previous study. Scanning electron microscope images of the fixed bacterium showed a rod‐shaped morphology. Initially the strain was named as *Pseudomonas* sp. sp. 102515 in our previous study (Fidan and Zhan 2019). Our bioinformatics and biochemical analysis in this study indicated that it is a novel *Pseudomonas* species and named as *Pseudomonas loganensis* sp. nov. to acknowledge the city of Logan, where the strain was discovered.

### 3.2 Phenotypic Characterization

The *P. loganensis* sp. nov. strain was characterized phenotypically by using the Biolog GENIII system. Carbon utilization assay showed that this strain can utilize a broad spectrum of carbon sources. Among these carbon sources, the top 10 carbon sources are l‐malic acid, thymidine, d‐ribose, d‐glucose 6‐phosphate, l‐serine, DL‐malic acid, acetic acid, d‐trehalose, d‐maltose, Gly‐Glu. The detailed lists of carbon sources and utilization profiles from Biolog was provided in Supporting Information S1: Table S1. Biolog results indicated that *P. loganensis* sp. nov. can have a high ability to adapt and grow under different carbon sources. The ability of wide range of substrate utilization can be advantageous to valorize different types of wastes, particularly pretreated ones (Elegbede et al. 2021). In addition, similar carbon sources utilization profiles was observed for other *Pseudomonas* species such as *Pseudomonas kairouanensis* sp. nov. and *Pseudomonas nabeulensis* sp. nov. (Oueslati et al. 2019).

Genome analysis using BlastKOALA KEGG Mapper revealed a wide range of consistent results regarding the carbon utilization profile of our isolate. Firstly, *Fru* family genes associated with fructose uptake and N_2_ regulation genes involved in nitrogen assimilation were identified as part of the phosphotransferase system (PTS) (Supporting Information S1: Figure S1). Notably, fructose utilization was confirmed through the carbon utilization assay. Secondly, KEGG Mapper analysis of ABC transporters (Supporting Information S1:Figure S2) indicated the presence of oligosaccharide, polyol, and lipid transporters. *P. loganensis* sp. nov. was found to possess transporters for sorbitol/mannitol, phospholipids, and nucleosides. The carbon utilization assay confirmed that our isolate could utilize sorbitol, mannitol, uridine, thymidine, adenosine, and inosine as carbon sources. Additionally, monosaccharide transporters for glucose/mannose, ribose, d‐xylose, l‐arabinose, and myo‐inositol were detected in the genome. Experimental results showed utilization of glucose, ribose, d‐xylose, l‐arabinose, and myo‐inositol, while growth on mannose was weaker than the negative control. Furthermore, genes for dipeptide transport were identified, and the isolate was confirmed to utilize Ala‐Gly, Gly‐Glu, and Gly‐Asp dipeptides as carbon sources.

Carbohydrate metabolism pathways analyzed via KEGG Mapper indicated the presence of complete gene sets for glycolysis, gluconeogenesis, pyruvate oxidation, the Krebs cycle, the pentose phosphate pathway, and the Entner‐Doudoroff pathway (Supporting Information S1: Figures S3–S5). Additional complete pathways included the De Ley‐Doudoroff pathway for d‐galactonate degradation, UDP‐glucose, UDP‐N‐acetyl‐d‐glucosamine, dTDP‐l‐rhamnose, undecaprenylphosphate alpha‐l‐Ara4N biosynthesis, and the methylcitrate cycle (Supporting Information S1: Figures S4, S6–S8). While genes for propanoate, butanoate, inositol phosphate, and C5‐branched dibasic acid metabolism were present, these pathways were incomplete. Genes for the glycine cleavage system and glyoxylate metabolism were also identified (Supporting Information S1: Figure S9). Starch and sucrose metabolism‐related genes suggested the ability for both biosynthesis and degradation of glycogen and trehalose (Supporting Information S1: Figure S10). Trehalose emerged as one of the most effective carbon sources in the carbon utilization assay. Among various enzymatic pathways for trehalose production, trehalose synthase (*treS*), known for directly converting maltose to trehalose (Song et al. 2016), was identified in the genome, highlighting the potential of this isolate for efficient trehalose production. Overall, there was strong consistency between genome analysis and the carbon utilization assay, as expected.

**Figure 1:**
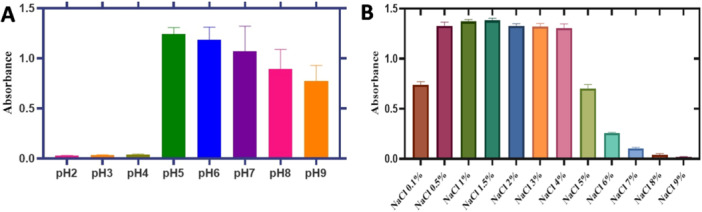
Growth profiles for *P. loganensis* sp. nov. at different pH (A) and NaCl concentrations (B).

pH tolerance test revealed that the bacterium exhibited a higher growth at pH 5. The growth rate slightly declined at pH 6 and 7, and progressively decreased under more alkaline conditions as the pH increased. There was no growth below pH 5 and significant reduction in growth rate at pH 9. pH 5 was determined to be the optimum for the growth of *P. loganensis* sp. nov. (Figure 1. A). On the other hand, the salinity tolerance tests demonstrated that *P. loganensis* sp. nov. had a wide tolerance range for NaCl concentration from 0.1% to ~5% NaCl. The strain grew efficiently across a broad NaCl concentration range (0.5%–4%) (Figure 1B). Furthermore, the genome of the bacterium includes genes encoding for Na^+^/H^+^ antiporter‐NhaA family and betaine aldehyde dehydrogenase, which are known to improve the salt tolerance (Fan et al. 2024). These findings indicated that the bacterium can tolerate NaCl concentrations from 0.1% to ~5% NaCl and different pH from 5 to 9. Since soil salinization damages agricultural lands, decreasing crop yield and productivity around the globe, stress‐tolerant plant growth‐promoting rhizobacteria (PGPR) can be applied in salt‐affected soils to remedy soil fertility (Priya et al. 2022). Previous studies investigated the impact of NaCl on the growth and PGPR activity of various *Pseudomonas* species (*P. aeruginosa*, *P. putida, P. cepacia*, *P. fluorescens*), revealing that none of these strains could survive above 3% NaCl (Deshwal and Kumar 2013). In addition, the co‐inoculation effect of *P. frederiksbergensis OB139* and *Pseudomonas vancouverensis OB155* improved the physiological properties of red pepper under salinity stress (Samaddar et al. 2019). Arora et. al. also reported a similar finding in their study, in which a salt‐tolerant *P. atacamensis* KSS‐6 along with organic manure improved the rice yield under salinity stress (Arora et al. 2024). Additionally, some *P. oryzihabitans* strains were reported to show a plant growth‐promoting effect for some different plants such as soybeans, pears, and tomatoes (Belimov et al. 2015; Cantabella et al. 2020; Kuzmicheva et al. 2017). Thus, our findings alongside with the literature suggests that *P. loganensis* sp. nov. could be a promising candidate for enhancing plant growth in saline and pH‐variable soils.

The colonies showed yellowish, small‐ to medium‐sized, circular, flat, and crusty morphology (Supporting Information S1: Figure S11). According to other biochemical tests (Table 1 and Supporting Information S1: Figure S11), only citrate utilization and lysine decarboxylation tests were positive, showing the bacterium can utilize citrate and decarboxylate lysine (Lal and Cheeptham 2015; MacWilliams 2009). The triple sugar iron agar test was negative because there was no explicit color change from red to black. This showed that the bacterium cannot produce hydrogen sulfide (Lehman 2005). Methyl red and Voges‐Proskauer tests and indole tests were negative, meaning that *P. loganensis* sp. nov. produce less acid from glucose fermentation, the butanediol fermentation pathway is not used and cannot degrade tryptophan to generate indole (MacWilliams 2012; McDevitt 2009). The blood agar test was also negative, indicating that the bacterium shows no β‐hemolysis (gamma hemolysis) (Buxton 2005). Moreover, the red color gradient across the motility medium indicated that this bacterium is motile (Shields and Cathcart 2011). The observed motility aligned with genomic findings, where flagellar proteins were identified (Table 5), suggesting an active motility mechanism. In Gram‐negative bacteria, the flagellum consists of three main components: the membrane complex, the hook, and the flagellin filament. The membrane complex anchors the flagellum to the cell surface, while the hook serves as a flexible linker between the rigid flagellin filament and the membrane complex. Filament rotation plays a crucial role in driving bacteria through liquid media, resulting in “swimming” motility (Bouteiller et al. 2021). In *Pseudomonas*, flagellar rotation generates a force that moves the cell body forward (Sampedro et al. 2015).

**Table 1:** Phenotypic characterization of *P. loganensis* sp. nov.

Biochemical Tests	Results
Simmons citrate agar	Positive, dark blue color
Triple sugar iron agar	Negative, no or less color change
Lysine decarboxylase broth	Positive, purple color
Methyl red	Negative, yellowish color
Voges‐Proskauer	Negative
Indole	Negative, yellowish color
Blood agar test	Negative, gamma hemolysis
Motility test	Positive, color change to red

### 3.3 Chemotaxonomic Analysis

Based on the interpretation of GC‐MS results, fatty acid methyl esters comprised 63.91% of all detected volatile compounds, as presented in Table 2. The remaining fractions consist of aliphatic alkanes, cyclic aliphatic aldehydes, aliphatic ethers, aliphatic cycloalcohols, nitriles, guanidine, and thiophene‐containing compounds. The membranes of the majority of *Pseudomonas* species include hydroxylated fatty acids characterized by the presence of C_10:0_ 3‐OH and C_12:0_ 2‐OH or C_12:1_ 3‐OH. This is a typical trait of this species (Moore et al. 2006; Palleroni 2005). Interestingly, *P. loganensis* sp. nov. exhibits 1,83% C_10:0_ 3‐OH and 0,88% C_12:0_ 1,2‐OH hydroxylated fatty acids on its cell membrane, confirming its membership in the *Pseudomonas* genus. On the other hand, the most abundant fatty acid on the cell membrane of *P. loganensis* sp. nov. was determined as C_36:0_ (47.3%) in triglyceride form. Other major fatty acids found in the cell membrane are C_19:1_ (8.14%), C_16:1_ (3.77%), C_13:1_ (1.26%), C_10:0_ (0.55%), and C_9:0_ (0.18%). Earlier studies showed that *P. aeruginosa*, *P. fluorescens*, *P. ovalis*, *P. acidovorans*, *P. diminuta*, *P. testosteroni*, and *P. lacunogenes* predominantly consists of straight‐chain C16:0, C16:1, and C18:1 fatty acids (Ikemoto et al. 1978). These fatty acids are key components of the inner membrane in most Gram‐negative bacteria. Additionally, hydroxylated fatty acids were absent in all Gram‐positive bacteria and thus they hold significant taxonomic value in the classification of Gram‐negative bacteria. C_10:0_ 3‐OH and C_12:0_ 1,2‐OH hydroxylated fatty acids of our isolate were similarly identified in many *Pseudomonas* strains (Ikemoto et al. 1978; Stead et al. 1992).

**Table 2:** Chemotaxonomic characterization of *Pseudomonas loganensis* sp. nov. through the analysis of cell membrane fatty acid content (%) using GC‐MS.

Fatty acid	Composition (%)
C_36:0_	47.3
C_19:1_	8.14
C_16:1_	3.77
C_13:1_	1.26
C_12:0_ 1,2‐OH	0.88
C_10:0_ 3‐OH	1.83
C_10:0_	0.55
C_9:0_	0.18
Total	63.91

### 3.4 Antibiogram Analysis

Based on the performance standards for antimicrobial disk susceptibility tests by Clinical and Laboratory Standards Institute (CLSI), this *P. loganensis* sp. nov. strain was found to be susceptible to kanamycin, azithromycin, tetracycline, and amikacin, while it was resistant to multiple antibiotics such as oxacillin, methicillin, penicillin G, ampicillin, and vancomycin (Table 3). Particularly, the highest zone of inhibition was observed for tetracycline. Additionally, *P. loganensis* sp. nov. exhibited intermediate susceptibility or dose‐dependent susceptibility against streptomycin (CLSI 2020). The genome of the bacterium was also analyzed via BlastKOALA KEGG mapper for certain antibiotic resistance‐related genes, and it was found the genome includes certain genes involving in antifolate resistance, β‐lactam resistance, cationic antimicrobial peptide (CAMP) resistance, insulin resistance, platinum drug resistance, and vancomycin resistance. Particularly, there are plenty of genes from the gene clusters for vancomycin, β‐lactam, and CAMP resistance. Complete gene sets for imipenem resistance and multidrug resistance were assigned in KEGG mapper. Antibiogram analysis confirmed these findings that *P. loganensis* sp. nov. is indeed resistant to vancomycin, ampicillin, methicillin, oxacillin, and penicillin G. In the literature, most *Pseudomonas* species, including *P. oryzihabitans* and *P. aeruginosa*, are known to be resistant to penicillin and the majority of related β‐lactam antibiotics. Notably, *P. oryzihabitans* has been reported to exhibit resistance to ampicillin similarly to our isolate (Hleba et al. 2014; Hur and Cho 2020).

**Table 3:** Antibiogram analysis of *P. loganensis* sp. nov. (R): resistant, (I): intermediate, (S): sensitive.

Antibiotics	Zone diameter (mm)
Oxacillin (OX‐1)	0 (R)
Methicillin (ME‐5)	0 (R)
Kanamycin (K‐30)	19.5 ± 0.25 (I)
Azithromycin (AZM‐15)	25.4 ± 0.08 (S)
Penicillin G (P‐10)	0 (R)
Ampicillin (AMP‐10)	0 (R)
Tetracycline (TE‐30)	30.6 ± 0.16 (S)
Vancomycin (VA‐30)	0 (R)
Amikacin (AK‐30)	19.9 ± 0.34 (I)
Streptomycin (S‐10)	17.9 ± 0.34 (I)

### 3.5 Phylogenetic Analysis, dDDh Interpretation, and Whole Genome ANI Comparison

**Figure 2:**
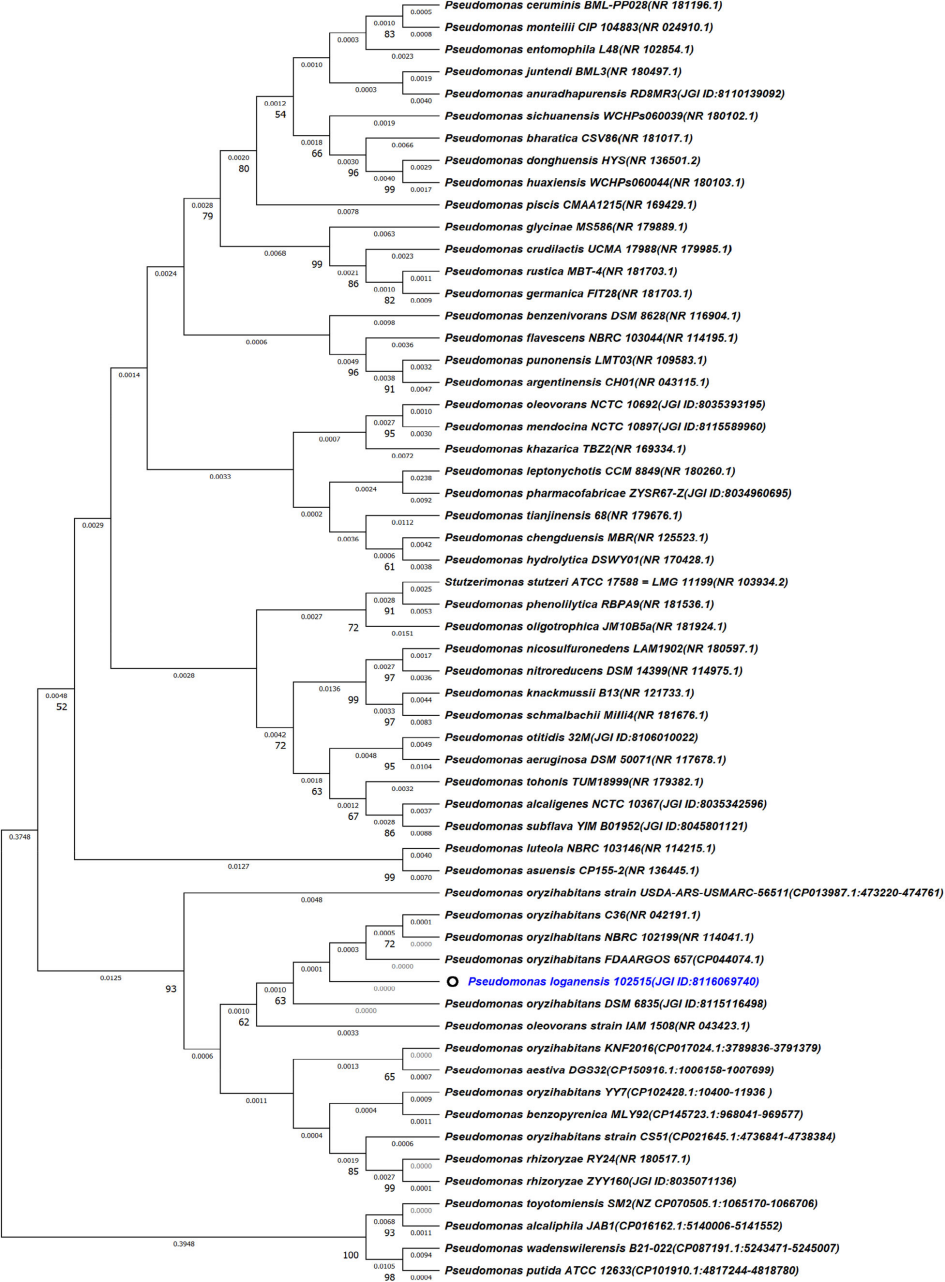
The phylogenetic tree is derived from the 16S rRNA genes of P. loganensis sp. nov. and other members that exhibit 95% or more similarity to it. Dendrograms were constructed using the neighbor‐joining method. Bootstrap frequencies exceeding 50% (which came from 1000 replicates) are shown at the nodes. Accession numbers or Identifiers are provided in parenthesis, sourced from NCBI and JGI‐IMG/M. The evolutionary distances were computed using the Maximum Composite Likelihood method and are in the units of the number of base substitutions per site. This analysis involved 58 nucleotide sequences. All ambiguous positions were removed for each sequence pair (pairwise deletion option). There were a total of 1850 positions in the final data set.

The dendrogram of 16S rRNA gene sequences from the selected strain of this study was generated (Figure 2). In this analysis, 58 different 16S rRNA sequences were evaluated and all ambiguous positions were removed for each sequence pair (because of pairwise deletion option). NCBI‐Blast analysis of 16S rRNA gene revealed that *P. loganensis* sp. nov. showed 100% sequence similarity with *Pseudomonas oryzihabitans* DSM 6835 and C36 (Supporting Information S1: Table S2). This finding was verified through Protologger assessment. Additionally *P. loganensis* sp. nov. were represented 99.94%, 99.93%, 99.74%, 99.68%, 99.68%, 99.41%, 99.349%, and 96.81% sequence similarity with *Pseudomonas oryzihabitans* FDAARGOS_657, *Pseudomonas oryzihabitans* NBRC 102199, *Pseudomonas oryzihabitans* LMG 7040, *Pseudomonas benzopyrenica* MLY92, *Pseudomonas oryzihabitans* KNF2016, *Pseudomonas oleovorans* IAM 1508, *Pseudomonas rhizoryzae* ZYY160, *Pseudomonas rhizoryzae* RY24, and *Stutzerimonas stutzeri* ATCC 17588, respectively. On the other hand, the 16S rRNA‐based evolutionary divergence matrix of *Pseudomonas* species was computed using the Maximum Composite Likelihood method, with evolutionary distances expressed as the number of base substitutions per site. A total of 1850 positions in the final data set were analyzed (Supporting Information S1: Table S3). Based on these findings, *P. loganensis* sp. nov. was shown zero evolutionary divergence with *Pseudomonas oryzihabitans* C36(NR_042191.1), *Pseudomonas oryzihabitans* NBRC102199(NR_114041.1), and *Pseudomonas oryzihabitans* DSM 6835 (JGI_ID:8115116498). In this issue, it appeared that the 16S rRNA does not have discriminatory capacity.

**Figure 3:**
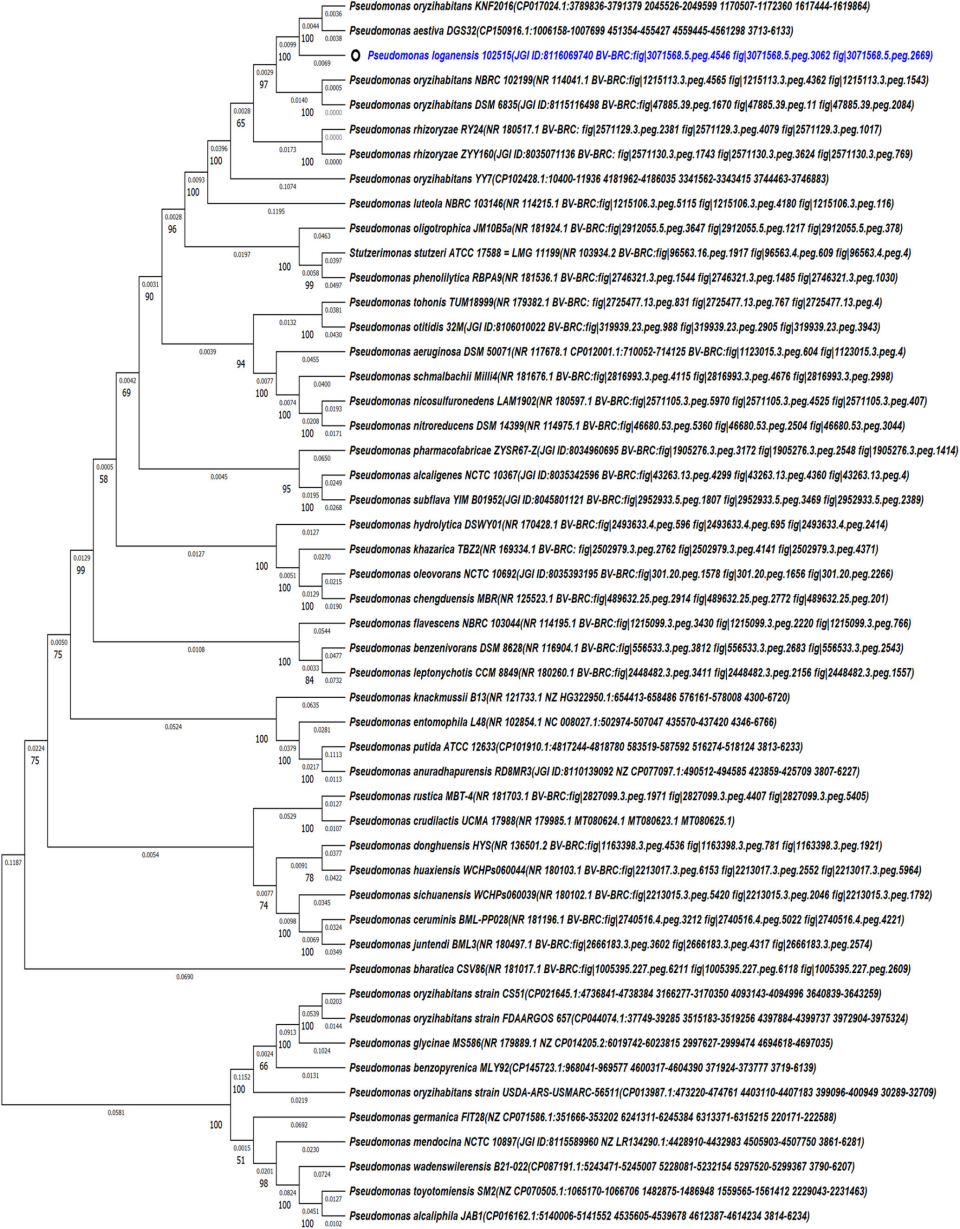
Phylogenetic tree based on concatenated sequences of 16S rRNA, *rpoB*, *rpoD*, and *gyrB* genes of *P. loganensis* sp. nov., *P. aestiva*, *P. oryzihabitans*, *P. rhizoryzae*, and other 95% and over nucleotide identity exhibiting *Pseudomonas* members in accordance with 16S rRNA. Branches corresponding to partitions reproduced in less than 50% bootstrap replicates are collapsed. The percentage of replicate trees in which the associated taxa clustered together in the bootstrap test (1000 replicates) are shown next to the branches. The evolutionary distances were computed using the Maximum Composite Likelihood method and are in the units of the number of base substitutions per site. This analysis involved 50 nucleotide sequences. All ambiguous positions were removed for each sequence pair (pairwise deletion option). There was a total of 11,792 positions in the final data set. Identifiers or accessions numbers belong to 16S rRNA, *rpoB*, *rpoD*, and *gyrB* genes that were acquired from NCBI, BV‐BRC, or JGI‐IMG/M and were presented in parentheses, respectively.

Before concatenation of *rpoB*, *rpoD*, and *gyrB* sequences with 16S rRNA sequences, all housekeeping genes screened against NCBI‐Blast database. According to the results, *P. loganensis* sp. nov. exhibited *rpoB* sequence similarity ranging between 97.79% and 96.61% with *P. aestiva*, *P. benzopyrenica*, and *P. oryzihabitans* (Supporting Information S1: Table S4). Furthermore, the similarities of *rpoD* gene sequences with *P. loganensis* sp. nov. differed between 99.19% to 96.66% which were also shown in Supporting Information S1: Table S4. On the other hand, sequence resemblence of *gyrB* genes with *P. loganensis* sp. nov. ranges between 98.60%–93.56% (Supporting Information S1: Table S4). The multilocus sequence analysis of the concatenated sequences of 16S rRNA, *rpoB*, *rpoD*, and *gyrB* housekeeping genes revealed that *P. oryzihabitans* KNF2016 and *P. aestiva* DGS32 showed 0.0148891934 and 0.0150922288 score of evolutionary divergence with *P. loganensis* sp. nov., which are the most close values among analyzed *Pseudomonas* members (Figure 3). Further, *P. oryzihabitans* DSM 6835, *P. oryzihabitans* NBRC102199, *P. rhizoryzae* ZYY160, and *P. rhizoryzae* RY24 strains represented 0.0301054366, 0.0303400647, 0.0395153775, and 0.0395317799 score of evolutionary discrepancy with *P. loganensis* sp. nov., correspondingly (Supporting Information S1: Table S5). Moreover, the phylogenetic tree constructed from the aforementioned concatenated data confirmed these findings. These findings underline the significance of ANI and POCPu comparison analysis based on whole genome sequence data.

*P. loganensis* sp. nov. genome was deposited to JGI‐IMG/M and an ANI analysis conducted in IMG/M. Based on the ANI results of IMG/M, *P. loganensis* showed singleton cluster type (ID:45310) among the *Pseudomonas* members found in 408 cliques. In addition, an another ANI analysis carried out independently using whole‐genome sequence data of *Pseudomonas* members from concatenated sequence phylogenetic tree. The whole genome sequences were chosen in accordance with evolutionary divergence scores from lowest to the highest. It was observed that *P. loganensis* sp. nov. was exhibited 94.56%, 93.92%, 93.91%, 92.99%, 89.51%, and 89.4% ANI similarity with *P. oryzihabitans* KNF2016, *P. oryzihabitans* NBRC 102199, *P. oryzihabitans* DSM 6835, *P. oryzihabitans* YY7, *P. rhizoryzae* ZYY160, and *P. rhizoryzae* RY24, respectively. On the other hand, the other remaining compared genomes displayed resemblance amongst 79.07% to 80.86% (Supporting Information S1: Figure S12). Besides, COG functional categories and their phylogenetic distribution were summarized in Table 4. Out of the 5368 total genes with COG assignment, 5237 genes demonstrated over 90% BLAST identity with *Pseudomonadota*, while only two genes exhibited more than 90% BLAST identity with *Actinomycetota*, associated with “energy production and conversion” and “general function prediction only.” Conversely, 129 genes linked to various COG categories were designated as unassigned to any particular phylum. These genes may be flanked with mobile genetic elements, such as genomic islands, transposons, or certain phage‐derived genes (Yetiman et al. 2024; Yetiman et al. 2022). Not only that, but *P. loganensis* showed POCPu values higher than 50% with all the *Pseudomonas* species found in the protologger databases (Supporting Information S1: Table S6). This also confirms that *P. loganensis*, sp. nov., belongs to the *Pseudomonas* genus (Qin et al. 2014). Besides, no species with a sequenced genome in the GTDB‐TK database exhibited an ANI value exceeding 95% when compared to the studied genome (Table 5). All of these evidence prove that *Psedumonas loganensis* sp. nov. is a putative novel *Psedumonas* species. Furthermore, the genome of *P. loganensis* sp. nov. was screened against MAGs from JGI‐IMG/MER and Protologger, but there is no match observed. Interestingly, Protologger's analysis did not identify any CRISPR arrays within the genome, which may influence the isolate's adaptive immunity against bacteriophages. Additionally, the genome's unique features (mentioned in Table 5) and lack of close relatives highlight its potential as a novel species for further functional and ecological studies.

**Table 4:** Phylogenetic distribution of genes linked to COG functional categories of *P. loganensis* sp. nov. between *Actinomycetota* and *Pseudomonadota* exhibiting above 90% BLAST identity.

COG Functional Category	*Actinomycetota*	*Pseudomonadota*	unassigned
Amino acid transport and metabolism	0	590 (11.3)	7 (5.4)
Carbohydrate transport and metabolism	0	407 (7.8)	6 (4.7)
Cell cycle control, cell division, chromosome partitioning	0	47 (0.9)	0
Cell motility	0	175 (3.3)	10 (7.8)
Cell wall/membrane/envelope biogenesis	0	274 (5.2)	9 (7.0)
Chromatin structure and dynamics	0	3 (0.1)	0
Coenzyme transport and metabolism	0	223 (4.3)	3 (2.3)
Defense mechanisms	0	82 (1.6)	0
Energy production and conversion	1 (50.0)	272 (5.2)	7 (5.4)
Extracellular structures	0	38 (0.7)	3 (2.3)
Function unknown	0	260 (5.0)	7 (5.4)
General function prediction only	1 (50.0)	569 (10.9)	13 (10.1)
Inorganic ion transport and metabolism	0	407 (7.8)	2 (1.6)
Intracellular trafficking, secretion, and vesicular transport	0	70 (1.3)	3 (2.3)
Lipid transport and metabolism	0	210 (4.0)	2 (1.6)
Mobilome: prophages, transposons	0	7 (0.1)	1 (0.8)
Nucleotide transport and metabolism	0	96 (1.8)	3 (2.3)
Posttranslational modification, protein turnover, chaperones	0	164 (3.1)	3 (2.3)
RNA processing and modification	0	0	0
Replication, recombination and repair	0	135 (2.6)	3 (2.3)
Secondary metabolites biosynthesis, transport, and catabolism	0	132 (2.5)	2 (1.6)
Signal transduction mechanisms	0	449 (8.6)	21 (16.3)
Transcription	0	367 (7.0)	12 (9.3)
Translation, ribosomal structure and biogenesis	0	259 (4.9)	12 (9.3)
Genes with no COG assignment		518	66
Total genes with COG assignment	2	5237	129
Total genes	1	4321	172
Percentage Median	50.0	3.1	2.3

*Note:* Numbers in () are COG pathways counts percentage.

**Table 5:** The descriptive summary is based on the Protologger evaluation results of *P. loganensis* sp. nov.

Descriptive feature	Result
16S rRNA analysis	The input 16S rRNA determined the isolate to be; not novel The best match was to; Pseudomonas oryzihabitans C36‐AJ575816‐ (100.0%)
Genome analysis	ANI identified the input genome as novel, with the best match being to Pseudomonas_oryzihabitans with a value of 93.9129% No valid species with a sequenced genome within the GTDB‐TK database was identified to have a ANI value > 95% with the studied genome The input genome belongs to the same genus as all database species, as determined by POCPu values > 50%
Matched MAGs	No metagenome‐assembled genomes (MAGs) that match *P. loganensis* sp. nov. were identified from Protologger and JGI‐IMG/MER.
Genome size	4.88 Mbp
G + C percentage	66.05%
Total genes	4575
Coding Sequences identified	4455
Protein coding sequences (CDSs)	4444
RNA genes	82
rRNAs	4, 1, 7 (5S, 16S, 23S)
tRNAs	61
ncRNAs	9
CRISPR arrays identified	0
Total pseudo genes	49
Pseudo genes (ambiguous residues)	0 of 49
Pseudo genes (frameshifted)	16 of 49
Pseudo genes (incomplete)	43 of 49
Pseudo genes (internal stop)	4 of 49
Pseudo genes (multiple problems)	13 of 49
Number of transporters	335
Number of secretion genes	82
Number of unique enzymes	1183
Presence of flagella	The following flagellar proteins were identified within the genome; *FlhA, FlhB, FlgB, FlgC, FlgD, FlgE, FlgF, FlgG, FlgH, FlgI, FlgJ, FlgK, FlgL, FliC, FliD, FliE, FliF, FliG, FliK, FliM, FliN, MotA, MotB, MotY*
Urease activity	The urease cluster (alpha, beta and gamma subunits) were identified within the genome (EC:3.5.1.5) Urea degradation was predicted by the presence of GMM:MF0085.
Number of predicted CAZymes	263
Predicted glycoside hydrolase families^a^	GH13 (15), GH77 (1), GH23 (8), GH1 (1), GH28 (2), GH10(1), GH101(1), GH16(3), GH35(1), GH94(1), GH4(1), GH0(9), GH73(3), GH43(1), GH3(2), GH8(1), GH103(3), GH135(1), GH65(1), GH33(1), GH36(1), GH5(7), GH71(1), GH19(1), GH114(2), GH39(1), GH105(1), GH32(2), GH15(3), GH6(2), GH20(1), GH37(1)
Predicted glycoside transferase families^a^	GT4(34), GT2(37), GT5(3), GT0(9), GT1(15), GT104(1), GT84(1), GT22(1), GT27(1), GT13(1), GT28(5), GT19(1), GT51(4), GT73(1), GT26(1), GT83(2), GT30(1), GT35(1), GT9(3), GT20(1)
Predicted polysaccharise lyase families^a^	PL7 (1)
Predicted carbohydrate esterase families^a^	CE14 (5), CE12 (5), CE4 (2), CE0 (1), CE11 (4), CE9 (2), CE16(1)
Predicted carbohydrate‐binding module (CBM) families^a^	CBM48(7), CBM50(19), CBM32(1), CBM41(9), CBM51(1), CBM13(4), CBM20(2), CBM5(1), CBM6(1)

a Numbers in parentheses are gene numbers.

Based on the previous studies, it was reported that consensus tree construction using 16S rRNA gene sequences extensively used for species identification and taxanomic purposes (Mulet et al. 2012). Besides, MLSA approach which is based on analysis of four housekeeping gene sequences (16S rRNA, *rpoB*, *rpoD*, and *gyrB*) has proved its reliability from point of view of *Pseudomonas* phylogeny classification and identification of new strains (Frasson et al. 2017). However, these approaches did not provide sufficient discriminatory power for the identification of novel species in this present study. Therefore, digital DNA‐DNA hybridization (dDDH) analysis was performed to further clarify the taxonomic position of *Pseudomonas loganensis* sp. nov. in relation to its closest relatives. The dDDH values between *P. loganensis* strains and reference genomes such as *P. oryzihabitans* KNF2016, *P. oryzihabitans* FDAARGOS_657, and *P. benzopyrenica* MLY92 ranged from 83.1% to 80.5%, with confidence intervals well above the 70% species delineation threshold (Supporting Information S1: Table S7). These results indicate a high degree of genomic relatedness to these species. However, the dDDH values with more distantly related *Pseudomonas* species, such as *P. rhizoryzae* RY24 and *P. otitidis* BC12, dropped below the 70% threshold, supporting the distinctiveness of *P. loganensis* sp. nov. The 70% dDDH threshold is widely accepted for prokaryotic species delineation (Meier‐Kolthoff et al. 2013, 2014). Notably, while dDDH values suggest close relatedness, the average nucleotide identity (ANI) values between *P. loganensis* sp. nov. and its closest relatives remain below the 95%–96% cutoff, which is the current gold standard for species definition (Richter and Rosselló‐Móra 2009; Yetiman et al. 2024; Yetiman and Ortakci 2023). This combination of high dDDH but sub‐threshold ANI values highlights the genomic distinctiveness of *P. loganensis* sp. nov., suggesting that this is a putative new *Pseudomonas* species within *Pseudomonas oryzihabitans* group.

The genome of *P. loganensis* sp. nov. was also analyzed using AntiSMASH to predict the biosynthetic gene clusters for the secondary metabolites. Biosynthetic gene cluster predictions revealed both siderophore and carotenoid gene clusters with high similarity confidence (Supporting Information S1: Figure S13). Frederiksenibactin, enterobactin, viobactin, and turnerbactin gene clusters were predicted as catecholate siderophores (Supporting Information S1: Figure S14), which are exported to external environments to form stable complexes with ferric iron (Batista et al. 2019; Moynié et al. 2019; Naka and Haygood 2023; Stow et al. 2021). The biosynthesis of turnerbactin was reported to contribute to the nitrogen fixation for the plants (Liu et al. 2017). In addition, the carotenoid gene cluster in the genome of *P. loganensis* sp. nov. was predicted (Supporting Information S1: Figure S15) and confirmed in our previous study. We found that engineered *P. loganensis* sp. nov. was an efficient producer of zeaxanthin diglucoside with a titer of 380 ± 12 mg/L (Fidan and Zhan 2019). Overall, these features of *P. loganensis* sp. nov. indicate that this strain can potentially be utilized for efficient production of carotenoids in industry as well as in agriculture as biofertilizer.

## 4 Conclusion

*P. loganensis* sp. nov., an endophytic bacterium isolated from *T. chinensis*, was investigated through biochemical test, antibiogram tests, and bioinformatics analysis to be characterized for a proper taxonomic identification. The biochemical tests revealed that this bacterium has tolerance to salt up to 4%–5% NaCl concentration, whereas its pH tolerance can be limited at pH values of 5–9. The fatty acid profile included certain fatty acids, known to be found in *Pseudomonas* species. Bioinformatics analysis showed that *P. loganensis* sp. nov. is a close relative to *Pseudomonas oryzihabitans* based on four housekeeping genes, yet the combination of high dDDH but sub‐threshold ANI values highlights the genomic distinctiveness. It was therefore identified as a novel species and named as *P. loganensis* sp. nov.

## Author Contributions

**Melisa Z. Karaman:** methodology, validation, visualization, writing – original draft, investigation, formal analysis, data curation. **Ahmet E. Yetiman:** conceptualization, methodology, validation, visualization, writing – original draft, formal analysis, data curation, investigation. **Jixun Zhan:** writing – review and editing, resources. **Ozkan Fidan:** conceptualization, writing – original draft, writing – review and editing, supervision, funding acquisition, resources.

## Ethics Statement

The authors have nothing to report.

## Conflicts of Interest

The authors declare no conflicts of interest.

## Supporting information


**Figure S1:** Phosphotransferase system related genes available in the genome of *P. loganensis* sp. nov.
**Figure S2:** ABC transporters related genes available in the genome of *P. loganensis* sp. nov.
**Figure S3:** Genes involving in the glycolysis and gluconeogenesis in the genome of *P. loganensis* sp. nov.
**Figure S4:** Genes involving in the Krebs cycle and methylcitrate cycle in the genome of *P. loganensis* sp. nov.
**Figure S5:** Genes involving in pentose phosphate and Entner‐Doudoroff pathways in the genome of *P. loganensis* sp. nov.
**Figure S6:** Genes involving in De Ley Doudoroff pathway for D‐galactonate degradation in the genome of *P. loganensis* sp. nov.
**Figure S7:** Genes involving in the biosynthesis of UDP‐glucose, UDP‐N‐acetyl‐D‐glucosamine, and undecaprenylphosphate alpha‐L‐Ara4N in the genome of *P. loganensis* sp. nov.
**Figure S8:** Genes involving in the biosynthesis of dTDP‐L‐rhamnose in the genome of *P. loganensis* sp. nov.
**Figure S9:** Genes involving in the glycine cleavage system and glyoxylate metabolism in the genome of *P. loganensis* sp. nov.
**Figure S10:** Genes involving in the starch and sucrose metabolism metabolism in the genome of *P. loganensis* sp. nov.
**Figure S11:** The images of a streak‐plate with *P. loganensis* sp. nov. and of biochemical tests.
**Figure S12:** The heatmap illustrates the average nucleotide identity (ANI) phylogram of *P. loganensis* sp. nov. and other Pseudomonas members that were detected pursuant to aforecited neighbor joining trees. The heatmap was generated according to the FastANI algorithm. Based on FastANI values, *P. loganensis* sp. nov. showed a singleton feature.
**Figure S13:** Overview of predicted biosynthetic gene clusters from the genome of *P. loganensis* sp. nov.
**Figure S14:** The details of siderophore biosynthetic gene cluster in the genome of *P. loganensis* sp. nov. and its comparison with known clusters.
**Figure S15:** The details of carotenoid biosynthetic gene cluster in the genome of *P. loganensis* sp. nov. and its comparison with known clusters.

**Table S1:** The detailed lists of carbon sources and utilization profiles from Biolog.
**Table S2:** Proximity of type strains exhibiting a similarity to *P. loganensis* sp. nov. based on 16S rRNA sequencing.
**Table S3:** Estimated of Evolutionary Divergence between 16S rRNA sequences of Pseudomonas members including Pseudomonas loganensis 102515. The number of base substitutions per site from between sequences are shown. Analyses were conducted using the Maximum Composite Likelihood model.
**Table S4:** NCBI‐BLAST similarity scores showing over 90% identity with Pseudomonas loganensis based on rpoB, rpoD, and gyrB genes.

**Table S5:** Estimated of evolutionary divergence between concotanated sequences of 16S rRNA, rpoB, rpoD, and gyrB genes belonging to Pseduomonas strains analyzed in this study.
**Table S6:** The comparison of the percentage of conserved proteins (POCP) between *P. loganensis* sp. nov. and other Pseudomonas species.
**Table S7:** The digital DNA‐DNA hybridization results of Pseudomonas loganensis sp. nov. and other closely related Pseudomonas members based on the MLSA scheme.

## Data Availability

The data that supports the findings of this study are available in the supporting material of this article.
