# Supplementary material for: Biochemical Characterization and Genome Analysis of Pseudomonas loganensis sp. nov., a Novel Endophytic Bacterium
Source: Microbiologyopen. 2025 Aug 13;14(4):e70051. doi: 10.1002/mbo3.70051 (PMC12344725; doi:10.1002/mbo3.70051)
Supplement: Supplementary file 1 — Figure S1: Phosphotransferase system related genes available in the genome of P. loganensis sp. nov. Figure S2: ABC transporters related genes available in the genome of P. loganensis sp. nov. Figure S3: Genes involving in the glycolysis and gluconeogenesis in the genome of P. loganensis sp. nov. Figure S4: Genes involving in the Krebs cycle and methylcitrate cycle in the genome of P. loganensis sp. nov. Figure S5: Genes involving in pentose phosphate and Entner‐Doudoroff pathways in the genome of P. loganensis sp. nov. Figure S6: Genes involving in De Ley Doudoroff pathway for D‐galactonate degradation in the genome of P. loganensis sp. nov. Figure S7: Genes involving in the biosynthesis of UDP‐glucose, UDP‐N‐acetyl‐D‐glucosamine, and undecaprenylphosphate alpha‐L‐Ara4N in the genome of P. loganensis sp. nov. Figure S8: Genes involving in the biosynthesis of dTDP‐L‐rhamnose in the genome of P. loganensis sp. nov. Figure S9: Genes involving in the glycine cleavage system and glyoxylate metabolism in the genome of P. loganensis sp. nov. Figure S10: Genes involving in the starch and sucrose metabolism metabolism in the genome of P. loganensis sp. nov. Figure S11: The images of a streak‐plate with P. loganensis sp. nov. and of biochemical tests. Figure S12: The heatmap illustrates the average nucleotide identity (ANI) phylogram of P. loganensis sp. nov. and other Pseudomonas members that were detected pursuant to aforecited neighbor joining trees. The heatmap was generated according to the FastANI algorithm. Based on FastANI values, P. loganensis sp. nov. showed a singleton feature. Figure S13: Overview of predicted biosynthetic gene clusters from the genome of P. loganensis sp. nov. Figure S14: The details of siderophore biosynthetic gene cluster in the genome of P. loganensis sp. nov. and its comparison with known clusters. Figure S15: The details of carotenoid biosynthetic gene cluster in the genome of P. loganensis sp. nov. and its comparison with known clusters. [file MBO3-14-e70051-s003.dot]

**Biochemical characterization and genome analysis of *Pseudomonas loganensis* sp. nov., a novel endophytic bacterium**

Melisa Z. Karaman^a^, Ahmet E. Yetiman^b^, Jixun Zhan^c^, Ozkan Fidan^a,*^

^a^ Department of Bioengineering, Faculty of Life and Natural Sciences, Abdullah Gul University, Kayseri, Turkiye, 38080

^b^ Department of Food Engineering Department, Faculty of Engineering, Erciyes University, Kayseri, Turkiye, 38080

^c^ Department of Biological Engineering, College of Engineering, Utah State University, Logan, UT, USA, 84322-4105

E-mail address: [ozkan.fidan@agu.edu.tr](mailto:ozkan.fidan@agu.edu.tr)

**Table S1.** The detailed lists of carbon sources and utilization profiles from Biolog.

| **Plate Type** | **Chemical** | **Difference in Average Height** | **Info** |
| --- | --- | --- | --- |
| PM01 | L-Malic acid | 0.589132249 | C-Source; carboxylic acid |
| PM01 | Thymidine | 0.574666427 | C-Source; nucleoside |
| PM01 | D-Ribose | 0.563357956 | C-Source; carbohydrate |
| PM01 | D-Glucose 6-phosphate | 0.562288308 | C-Source; carbohydrate |
| PM01 | L-Serine | 0.561853836 | C-Source; amino acid |
| PM01 | DL-Malic acid | 0.533524401 | C-Source; carboxylic acid |
| PM01 | Acetic acid | 0.522485727 | C-Source; carboxylic acid |
| PM01 | D-Trehalose | 0.519722503 | C-Source; carbohydrate |
| PM01 | D-Maltose | 0.513145346 | C-Source; carbohydrate |
| PM01 | Gly-Glu | 0.512895745 | C-Source; peptide |
| PM01 | Pyruvic acid | 0.512591393 | C-Source; carboxylic acid |
| PM01 | N-Acetyl-D-Glucosamine | 0.510571937 | C-Source; carbohydrate |
| PM01 | alpha-D-Glucose | 0.507489914 | C-Source; carbohydrate |
| PM01 | Maltotriose | 0.505909941 | C-Source; carbohydrate |
| PM01 | Adenosine | 0.503790306 | C-Source; nucleoside |
| PM01 | L-Lactic acid | 0.501846058 | C-Source; carboxylic acid |
| PM01 | D-Cellobiose | 0.498631361 | C-Source; carbohydrate |
| PM01 | Propionic acid | 0.498256155 | C-Source; carboxylic acid |
| PM01 | D-Serine | 0.486280384 | C-Source; amino acid |
| PM01 | alpha-Keto-Glutaric acid | 0.482342143 | C-Source; carboxylic acid |
| PM01 | Fumaric acid | 0.481491604 | C-Source; carboxylic acid |
| PM01 | Inosine | 0.481124515 | C-Source; nucleoside |
| PM01 | Citric acid | 0.478427687 | C-Source; carboxylic acid |
| PM01 | Methyl Pyruvate | 0.476430091 | C-Source; ester |
| PM01 | 2`-Deoxyadenosine | 0.471816789 | C-Source; carbohydrate |
| PM01 | Ala-Gly | 0.469453243 | C-Source; peptide |
| PM01 | Acetoacetic acid | 0.468760591 | C-Source; carboxylic acid |
| PM01 | L-Glutamic acid | 0.465692258 | C-Source; amino acid |
| PM01 | DL-alpha-Glycerol phosphate | 0.462703006 | C-Source; carbohydrate |
| PM01 | D-Fructose 6-phosphoric acid | 0.460830281 | C-Source; carbohydrate |
| PM01 | D-Gluconic acid | 0.458919798 | C-Source; carboxylic acid |
| PM01 | Bromosuccinic acid | 0.454167617 | C-Source; carboxylic acid |
| PM01 | D-Fructose | 0.45353301 | C-Source; carbohydrate |
| PM01 | Uridine | 0.452796371 | C-Source; nucleoside |
| PM01 | L-Aspartic acid | 0.450788333 | C-Source; amino acid |
| PM01 | Formic acid | 0.44414509 | C-Source; carboxylic acid |
| PM01 | L-Threonine | 0.438620584 | C-Source; amino acid |
| PM01 | Glycerol | 0.437009616 | C-Source; carbohydrate |
| PM01 | L-Alanine | 0.435885777 | C-Source; amino acid |
| PM01 | Gly-Asp | 0.427964235 | C-Source; peptide |
| PM01 | L-Lyxose | 0.425221244 | C-Source; carbohydrate |
| PM01 | D-Xylose | 0.421191973 | C-Source; carbohydrate |
| PM01 | L-Asparagine | 0.414118872 | C-Source; amino acid |
| PM01 | DL-alpha-Hydroxy-Butyric acid | 0.409594982 | C-Source; carboxylic acid |
| PM01 | Methyl beta-D-Glucoside | 0.408152424 | C-Source; carbohydrate |
| PM01 | L-Proline | 0.386646114 | C-Source; amino acid |
| PM01 | D-Aspartic acid | 0.379376427 | C-Source; amino acid |
| PM01 | 2-Phenylethylamine | 0.376218641 | C-Source; amine |
| PM01 | Succinic acid monomethyl ester | 0.365914119 | C-Source; ester |
| PM01 | Tween 40 | 0.363607537 | C-Source; fatty acid |
| PM01 | N-Acetyl-D-Mannosamine | 0.361273871 | C-Source; carbohydrate |
| PM01 | Lactose | 0.355599593 | C-Source; carbohydrate |
| PM01 | D-Alanine | 0.349793631 | C-Source; amino acid |
| PM01 | D-Malic acid | 0.349697734 | C-Source; carboxylic acid |
| PM01 | D-Melibiose | 0.345548319 | C-Source; carbohydrate |
| PM01 | D-Mannitol | 0.344597915 | C-Source; carbohydrate |
| PM01 | L-Glutamine | 0.343092007 | C-Source; amino acid |
| PM01 | Sucrose | 0.341411361 | C-Source; carbohydrate |
| PM01 | alpha-Hydroxy-Glutaric acid g-lactone | 0.340766485 | C-Source; carboxylic acid |
| PM01 | Adonitol | 0.338900915 | C-Source; carbohydrate |
| PM01 | L-Arabinose | 0.338828603 | C-Source; carbohydrate |
| PM01 | Methyl alpha-D-Galactoside | 0.338337557 | C-Source; carbohydrate |
| PM01 | Tyramine | 0.336656941 | C-Source; amine |
| PM01 | Glycolic acid | 0.336458251 | C-Source; carboxylic acid |
| PM01 | D-Threonine | 0.335663609 | C-Source; amino acid |
| PM01 | Succinic acid | 0.333121646 | C-Source; carboxylic acid |
| PM01 | Mucic acid | 0.332916885 | C-Source; carboxylic acid |
| PM01 | 1,2-Propanediol | 0.332432493 | C-Source; alcohol |
| PM01 | Lactulose | 0.330770481 | C-Source; carbohydrate |
| PM01 | D-Psicose | 0.328033075 | C-Source; carbohydrate |
| PM01 | L-Rhamnose | 0.327503695 | C-Source; carbohydrate |
| PM01 | Gly-Pro | 0.325727957 | C-Source; peptide |
| PM01 | Tricarballylic acid | 0.323762403 | C-Source; carboxylic acid |
| PM01 | Dulcitol | 0.323715009 | C-Source; carbohydrate |
| PM01 | meso-Tartaric acid | 0.320613579 | C-Source; carboxylic acid |
| PM01 | D-Galacturonic acid | 0.317109318 | C-Source; carboxylic acid |
| PM01 | L-Fucose | 0.313220591 | C-Source; carbohydrate |
| PM01 | alpha-D-Glucose 1-phosphate | 0.312167381 | C-Source; carbohydrate |
| PM01 | 2-Aminoethanol | 0.310145697 | C-Source; alcohol |
| PM01 | L-Galactonic acid gamma-Lactone | 0.308534537 | C-Source; carboxylic acid |
| PM01 | D-Sorbitol | 0.308296145 | C-Source; carbohydrate |
| PM01 | myo-Inositol | 0.302478571 | C-Source; carbohydrate |
| PM01 | D-Saccharic acid | 0.301884273 | C-Source; carboxylic acid |
| PM01 | D-Galactose | 0.300624133 | C-Source; carbohydrate |
| PM01 | D-Glucuronic acid | 0.297552502 | C-Source; carboxylic acid |
| PM01 | Tween 80 | 0.295832324 | C-Source; fatty acid |
| PM01 | D-Galactonic acid gamma-lactone | 0.292139058 | C-Source; carboxylic acid |
| PM01 | alpha-Keto-Butyric acid | 0.290000077 | C-Source; carboxylic acid |
| PM01 | D-Glucosaminic acid | 0.288572653 | C-Source; carboxylic acid |
| PM01 | Glucuronamide | 0.287648862 | C-Source; amide |
| PM01 | Negative Control | 0.28681388 | C-Source; negative control |
| PM01 | D-Mannose | 0.249654926 | C-Source; carbohydrate |
| PM01 | m-Hydroxyphenyl Acetic acid | 0.248815921 | C-Source; carboxylic acid |
| PM01 | p-Hydroxyphenyl Acetic acid | 0.239380957 | C-Source; carboxylic acid |
| PM01 | Glyoxylic acid | 0.22305341 | C-Source; carboxylic acid |
| PM01 | Tween 20 | 0.220499007 | C-Source; fatty acid |

**Table S2.** Proximity of type strains exhibiting a similarity to *P. loganensis* sp. nov. based on 16S rRNA sequencing.

| **Species/Strain** | **Identity** | **Source** | **Gene ID**  **/Accession** |
| --- | --- | --- | --- |
| *Pseudomonas oryzihabitans* DSM 6835 | 100% | JGI-IMG/M | 8115116498 |
| *Pseudomonas oryzihabitans* C36 | 100% | NCBI | NR_042191.1 |
| *Pseudomonas oryzihabitans* NBRC 102199 | 99.93% | NCBI | NR_114041.1 |
| *Pseudomonas oryzihabitans* FDAARGOS_657 | 99.94% | NCBI | CP044074.1 |
| *Stutzerimonas stutzeri* ATCC 17588 | 96.81% | NCBI | NR_103934.2 |
| *Pseudomonas oryzihabitans* LMG 7040 | 99.74% | NCBI | NR_117269.1 |
| *Pseudomonas benzopyrenica* MLY92 | 99.68% | NCBI | CP145723.1 |
| *Pseudomonas oryzihabitans* KNF2016 | 99.68% | NCBI | CP017024.1 |
| *Pseudomonas oleovorans* IAM 1508 | 99.41% | NCBI | NR_043423.1 |
| *Pseudomonas rhizoryzae* ZYY160 | 99.349% | JGI-IMG/M | 8035071136 |
| *Pseudomonas rhizoryzae* RY24 | 99.35% | NCBI | NR_180517.1 |

**Table S4.** NCBI-BLAST similarity scores showing over 90% identity with *Pseudomonas loganensis* based on *rpoB*, *rpoD*, and *gyrB* genes.

| **Species/Strain** | **Gene** | **Identity** | **Gene ID**  **/Accession** |
| --- | --- | --- | --- |
| *Pseudomonas aestiva* DGS32 | *rpoB* | 97.79% | CP150916.1 |
| *Pseudomonas oryzihabitans* KNF2016 | *rpoB* | 97.74% | CP017024.1 |
| *Pseudomonas oryzihabitans* MS8 | *rpoB* | 97.57% | CP022198.1 |
| *Pseudomonas benzopyrenica* MLY92 | *rpoB* | 97.15% | CP145723.1 |
| *Pseudomonas oryzihabitans* FDAARGOS_657 | *rpoB* | 97.05% | CP044074.1 |
| *Pseudomonas oryzihabitans* YY7 | *rpoB* | 97.05% | CP102428.1 |
| *Pseudomonas oryzihabitans* USDA-ARS-USMARC-56511 | *rpoB* | 96.78% | CP013987.1 |
| *Pseudomonas oryzihabitans* GXM4 | *rpoB* | 96.74% | OR756234.1 |
| *Pseudomonas oryzihabitans* CS51 | *rpoB* | 96.61% | CP021645.1 |
| Pseudomonas oryzihabitans KNF2016 | *rpoD* | 99.19% | CP017024.1 |
| *Pseudomonas aestiva* DGS32 | *rpoD* | 99.14% | CP150916.1 |
| *Pseudomonas oryzihabitans* MS8 | *rpoD* | 98.92% | CP022198.1 |
| *Pseudomonas oryzihabitans* YY7 | *rpoD* | 97.57% | CP102428.1 |
| *Pseudomonas benzopyrenica* MLY92 | *rpoD* | 97.14% | CP145723.1 |
| *Pseudomonas oryzihabitans* USDA-ARS-USMARC-56511 | *rpoD* | 97.09% | CP013987.1 |
| *Pseudomonas oryzihabitans* CS51 | *rpoD* | 96.71% | CP021645.1 |
| *Pseudomonas oryzihabitans* GXM4 | *rpoD* | 96.66% | OR735508.1 |
| *Pseudomonas oryzihabitans* KNF2016 | *gyrB* | 98.60% | CP017024.1 |
| *Pseudomonas aestiva* DGS32 | *gyrB* | 98.51% | CP150916.1 |
| *Pseudomonas oryzihabitans* MS8 | *gyrB* | 98.06% | CP022198.1 |
| *Pseudomonas oryzihabitans* USDA-ARS-USMARC-56511 | *gyrB* | 95.33% | CP013987.1 |
| *Pseudomonas benzopyrenica* MLY92 | *gyrB* | 95.04% | CP145723.1 |
| *Pseudomonas oryzihabitans* YY7 | *gyrB* | 94.93% | CP102428.1 |
| *Pseudomonas oryzihabitans* FDAARGOS_657 | *gyrB* | 94.59% | CP044074.1 |
| *Pseudomonas oryzihabitans* GXM4 | *gyrB* | 93.61% | OR735509.1 |
| *Pseudomonas oryzihabitans* CS51 | *gyrB* | 93.56% | CP021645.1 |

| **Table S6.** The comparison of the percentage of conserved proteins (POCP) between *P. loganensis* sp. nov. and other *Pseudomonas* species. | |
| --- | --- |
| #Matching species | POCPu (%) |
| POCPu suggests the input genome and Pseudomonas_oryzihabitans--RS_GCF_000730625.1 belong to the same genus with a POCPu was; | 0.931240 |
| POCPu suggests the input genome and Pseudomonas_rhizoryzae--RS_GCF_005250615.1 belong to the same genus with a POCPu was; | 0.863048 |
| POCPu suggests the input genome and Pseudomonas_luteola--RS_GCF_002091675.1 belong to the same genus with a POCPu was; | 0.679879 |
| POCPu suggests the input genome and Pseudomonas_punonensis--RS_GCF_900142655.1 belong to the same genus with a POCPu was; | 0.655471 |
| POCPu suggests the input genome and Pseudomonas_argentinensis--RS_GCF_900113905.1 belong to the same genus with a POCPu was; | 0.647830 |
| POCPu suggests the input genome and Pseudomonas_flavescens--RS_GCF_900100535.1 belong to the same genus with a POCPu was; | 0.622247 |
| POCPu suggests the input genome and Pseudomonas_khorasanensis--RS_GCF_014268505.2 belong to the same genus with a POCPu was; | 0.613349 |
| POCPu suggests the input genome and Pseudomonas_hamedanensis--RS_GCF_014268595.2 belong to the same genus with a POCPu was; | 0.611911 |
| POCPu suggests the input genome and Pseudomonas_urmiensis--RS_GCF_014268815.2 belong to the same genus with a POCPu was; | 0.607016 |
| POCPu suggests the input genome and Pseudomonas_farsensis--RS_GCF_014268805.2 belong to the same genus with a POCPu was; | 0.606247 |
| POCPu suggests the input genome and Pseudomonas_atacamensis--RS_GCF_004801935.1 belong to the same genus with a POCPu was; | 0.603810 |
| POCPu suggests the input genome and Pseudomonas_glycinae--RS_GCF_001594225.2 belong to the same genus with a POCPu was; | 0.600748 |
| POCPu suggests the input genome and Pseudomonas_siliginis--RS_GCF_019145195.1 belong to the same genus with a POCPu was; | 0.598492 |
| POCPu suggests the input genome and Pseudomonas_reidholzensis--RS_GCF_900536025.1 belong to the same genus with a POCPu was; | 0.593649 |
| POCPu suggests the input genome and Pseudomonas_tensinigenes--RS_GCF_014268445.2 belong to the same genus with a POCPu was; | 0.593229 |
| POCPu suggests the input genome and Pseudomonas_gozinkensis--RS_GCF_014863585.1 belong to the same genus with a POCPu was; | 0.592549 |
| POCPu suggests the input genome and Pseudomonas_inefficax--RS_GCF_900277125.1 belong to the same genus with a POCPu was; | 0.592523 |
| POCPu suggests the input genome and Pseudomonas_vanderleydeniana--RS_GCF_014268755.2 belong to the same genus with a POCPu was; | 0.591705 |
| POCPu suggests the input genome and Pseudomonas_muyukensis--RS_GCF_019139535.1 belong to the same genus with a POCPu was; | 0.587604 |
| POCPu suggests the input genome and Pseudomonas_kermanshahensis--RS_GCF_014269205.2 belong to the same genus with a POCPu was; | 0.587047 |
| POCPu suggests the input genome and Pseudomonas_fakonensis--RS_GCF_019139895.1 belong to the same genus with a POCPu was; | 0.586440 |
| POCPu suggests the input genome and Pseudomonas_maumuensis--RS_GCF_019139675.1 belong to the same genus with a POCPu was; | 0.584746 |
| POCPu suggests the input genome and Pseudomonas_juntendi--RS_GCF_009932375.1 belong to the same genus with a POCPu was; | 0.584208 |
| POCPu suggests the input genome and Pseudomonas_aeruginosa--RS_GCF_001457615.1 belong to the same genus with a POCPu was; | 0.583716 |
| POCPu suggests the input genome and Pseudomonas_alcaliphila--RS_GCF_900101755.1 belong to the same genus with a POCPu was; | 0.582671 |
| POCPu suggests the input genome and Pseudomonas_xantholysinigenes--RS_GCF_014268885.2 belong to the same genus with a POCPu was; | 0.581984 |
| POCPu suggests the input genome and Pseudomonas_shirazensis--RS_GCF_014268785.2 belong to the same genus with a POCPu was; | 0.580493 |
| POCPu suggests the input genome and Pseudomonas_mendocina--RS_GCF_900636545.1 belong to the same genus with a POCPu was; | 0.579090 |
| POCPu suggests the input genome and Pseudomonas_khazarica--RS_GCF_004521985.1 belong to the same genus with a POCPu was; | 0.576703 |
| POCPu suggests the input genome and Pseudomonas_asiatica--RS_GCF_009932335.1 belong to the same genus with a POCPu was; | 0.576651 |
| POCPu suggests the input genome and Pseudomonas_allokribbensis--RS_GCF_014863605.1 belong to the same genus with a POCPu was; | 0.575344 |
| POCPu suggests the input genome and Pseudomonas_laurylsulfatiphila--RS_GCF_002934665.1 belong to the same genus with a POCPu was; | 0.573921 |
| POCPu suggests the input genome and Pseudomonas_toyotomiensis--RS_GCF_900115695.1 belong to the same genus with a POCPu was; | 0.569313 |
| POCPu suggests the input genome and Pseudomonas_chengduensis--RS_GCF_900102635.1 belong to the same genus with a POCPu was; | 0.565373 |
| POCPu suggests the input genome and Pseudomonas_peradeniyensis--RS_GCF_014268935.2 belong to the same genus with a POCPu was; | 0.564643 |
| POCPu suggests the input genome and Pseudomonas_wadenswilerensis--RS_GCF_900497695.1 belong to the same genus with a POCPu was; | 0.562835 |
| POCPu suggests the input genome and Pseudomonas_otitidis--RS_GCF_900111835.1 belong to the same genus with a POCPu was; | 0.561808 |
| POCPu suggests the input genome and Pseudomonas_tohonis--RS_GCF_012767755.2 belong to the same genus with a POCPu was; | 0.559430 |
| POCPu suggests the input genome and Pseudomonas_sichuanensis--RS_GCF_003231305.1 belong to the same genus with a POCPu was; | 0.557000 |
| POCPu suggests the input genome and Pseudomonas_benzenivorans--RS_GCF_900100495.1 belong to the same genus with a POCPu was; | 0.541992 |
| POCPu suggests the input genome and Pseudomonas_oleovorans--RS_GCF_002197815.1 belong to the same genus with a POCPu was; | 0.538174 |
| POCPu suggests the input genome and Pseudomonas_guangdongensis--RS_GCF_900105885.1 belong to the same genus with a POCPu was; | 0.512929 |

**Table S7.** The digital DNA-DNA hybridization results of *Pseudomonas loganensis* sp. nov. and other closely related Pseudomonas members based on the MLSA scheme.

| **Query genome** | **Reference genome** | **DDH** | **Model C.I.** | **Distance** | **Prob. DDH >= 70%** | **G+C difference** |
| --- | --- | --- | --- | --- | --- | --- |
| *Pseudomonas loganensis* sp. nov*.* | *Pseudomonas oryzihabitans KNF2016* | 83.10 | [79.3 - 86.4%] | 0.1165 | 93.73 | 0.06 |
| *Pseudomonas loganensis* sp. nov*.* | *Pseudomonas oryzihabitans FDAARGOS_657* | 82.10 | [78.2 - 85.4%] | 0.1219 | 92.91 | 0.19 |
| *Pseudomonas loganensis* sp. nov*.* | *Pseudomonas benzopyrenica MLY92* | 80.50 | [76.6 - 83.9%] | 0.1299 | 91.51 | 0.59 |
| *Pseudomonas loganensis* sp. nov*.* | *Pseudomonas oryzihabitans MS8* | 78.50 | [74.5 - 82%] | 0.1398 | 89.41 | 0.56 |
| *Pseudomonas loganensis* sp. nov*.* | *Pseudomonas psychrotolerans DSM 15758* | 70.90 | [66.9 - 74.5%] | 0.1788 | 76.43 | 0.74 |
| *Pseudomonas loganensis* sp. nov*.* | *Pseudomonas rhizoryzae RY24* | 67.20 | [63.3 - 70.8%] | 0.1988 | 66.52 | 1.23 |
| *Pseudomonas loganensis* sp. nov*.* | *Pseudomonas otitidis BC12* | 18.80 | [15.7 - 22.4%] | 0.7446 | 0 | 1.23 |
| *Pseudomonas loganensis* sp. nov*.* | *Pseudomonas oligotrophica JM10B5a* | 18.60 | [15.5 - 22.1%] | 0.7525 | 0 | 1.12 |
| *Pseudomonas loganensis* sp. nov*.* | *Pseudomonas tohonis TUM18999* | 18.40 | [15.3 - 22%] | 0.7581 | 0 | 0.31 |
| *Pseudomonas loganensis* sp. nov*.* | *Pseudomonas aeruginosa DSM 50071 = NBRC 12689* | 18.30 | [15.2 - 21.9%] | 0.7599 | 0 | 0.43 |
| *Pseudomonas loganensis* sp. nov*.* | *Pseudomonas alcaligenes NCTC10367* | 18.20 | [15.1 - 21.8%] | 0.7637 | 0 | 1.55 |
| *Pseudomonas loganensis* sp. nov*.* | *Pseudomonas schmalbachii Milli4* | 18.00 | [14.9 - 21.5%] | 0.7703 | 0 | 1.1 |
| *Pseudomonas loganensis* sp. nov*.* | *Pseudomonas khazarica TBZ2* | 17.90 | [14.8 - 21.4%] | 0.7743 | 0 | 1.11 |
| *Pseudomonas loganensis* sp. nov*.* | *Pseudomonas nitroreducens DSM 14399* | 17.70 | [14.6 - 21.2%] | 0.7802 | 0 | 1.05 |
| *Pseudomonas loganensis* sp. nov*.* | *Pseudomonas nicosulfuronedens LAM 1902* | 17.50 | [14.5 - 21.1%] | 0.7847 | 0 | 1.27 |
| *Pseudomonas loganensis* sp. nov*.* | *Pseudomonas flavescens NBRC103044* | 17.10 | [14 - 20.6%] | 0.8007 | 0 | 2.57 |
| *Pseudomonas loganensis* sp. nov*.* | *Pseudomonas sichuanensis WCHPs060039* | 17.10 | [14.1 - 20.6%] | 0.7990 | 0 | 2.19 |
| *Pseudomonas loganensis* sp. nov*.* | *Pseudomonas benzenivorans DSM 8628* | 17.00 | [14 - 20.5%] | 0.8029 | 0 | 0.84 |
| *Pseudomonas loganensis* sp. nov*.* | *Pseudomonas chengduensis WD211* | 16.90 | [13.9 - 20.5%] | 0.8054 | 0 | 3.3 |
| *Pseudomonas loganensis* sp. nov*.* | *Pseudomonas toyotomiensis SM2* | 16.90 | [13.9 - 20.5%] | 0.8049 | 0 | 3.28 |
| *Pseudomonas loganensis* sp. nov*.* | *Pseudomonas oleovorans GD04132* | 16.80 | [13.8 - 20.4%] | 0.8088 | 0 | 3.74 |
| *Pseudomonas loganensis* sp. nov*.* | *Pseudomonas alcaliphila JAB1* | 16.80 | [13.8 - 20.3%] | 0.8106 | 0 | 3.51 |
| *Pseudomonas loganensis* sp. nov*.* | *Pseudomonas chengduensis MBR* | 16.70 | [13.7 - 20.2%] | 0.8134 | 0 | 3.73 |
| *Pseudomonas loganensis* sp. nov*.* | *Pseudomonas wadenswilerensis B21-022* | 16.70 | [13.7 - 20.2%] | 0.8137 | 0 | 3.52 |
| *Pseudomonas loganensis* sp. nov*.* | *Pseudomonas songnenensis NEAU-ST5-5* | 16.30 | [13.4 - 19.8%] | 0.8267 | 0 | 2.8 |
| *Pseudomonas loganensis* sp. nov*.* | *Pseudomonas huaxiensis WCHPs060044* | 15.90 | [12.9 - 19.3%] | 0.8448 | 0 | 3.79 |
| *Pseudomonas loganensis* sp. nov*.* | *Pseudomonas glycinae MS586* | 15.40 | [12.5 - 18.9%] | 0.8623 | 0 | 5.57 |
| *Pseudomonas loganensis* sp. nov*.* | *Pseudomonas crudilactis UCMA 17988* | 14.60 | [11.8 - 18%] | 0.8958 | 0 | 6.92 |
| *Pseudomonas loganensis* sp. nov*.* | *Pseudomonas luteola NCTC11842* | 14.10 | [11.3 - 17.5%] | 0.9179 | 0 | 11.28 |
| *Pseudomonas loganensis* sp. nov*.* | *Pseudomonas duriflava CGMCC 1.6858* | 13.90 | [11.1 - 17.3%] | 0.9275 | 0 | 11.9 |


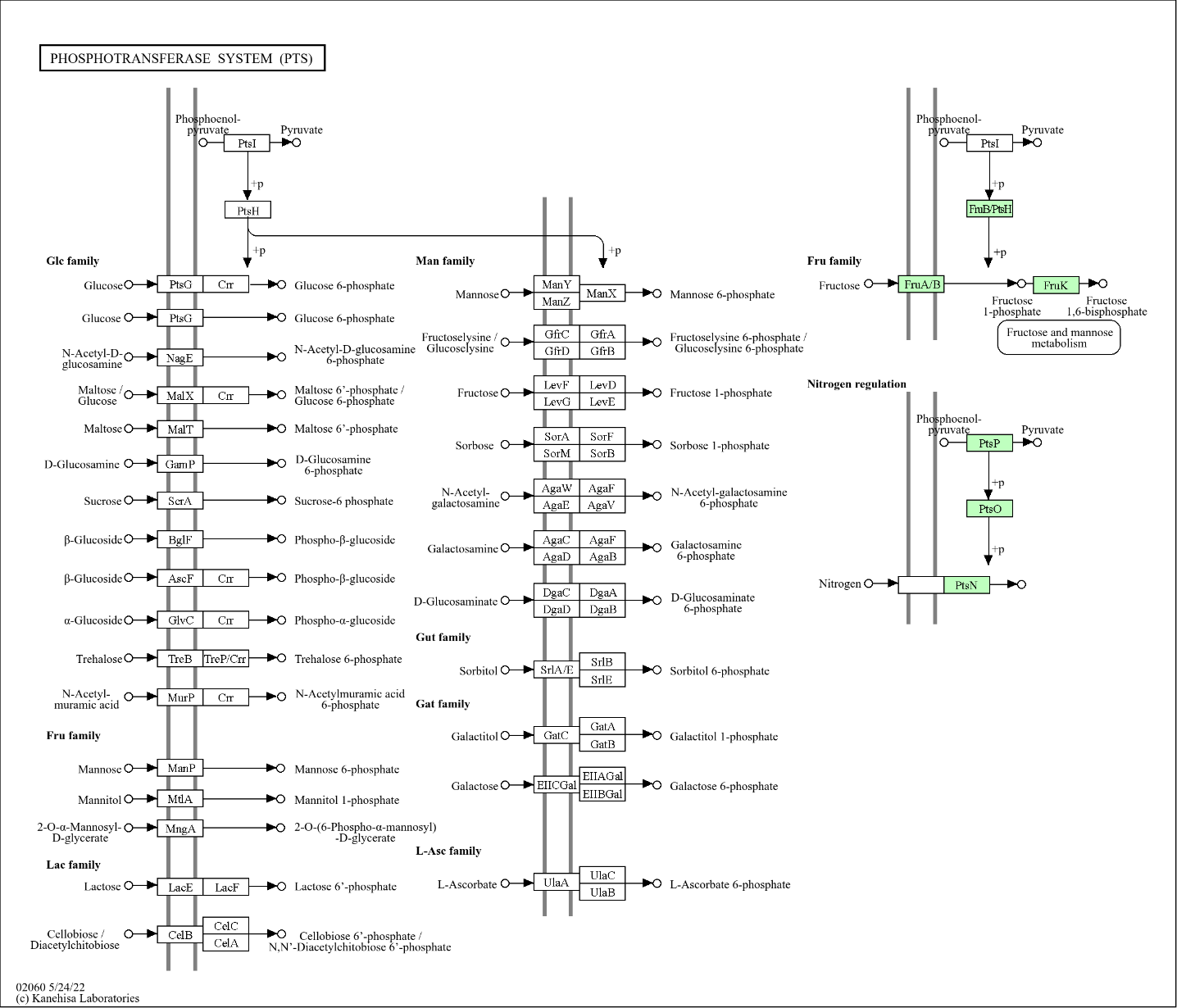


Figure S1. Phosphotransferase system related genes available in the genome of P. loganensis sp. nov.


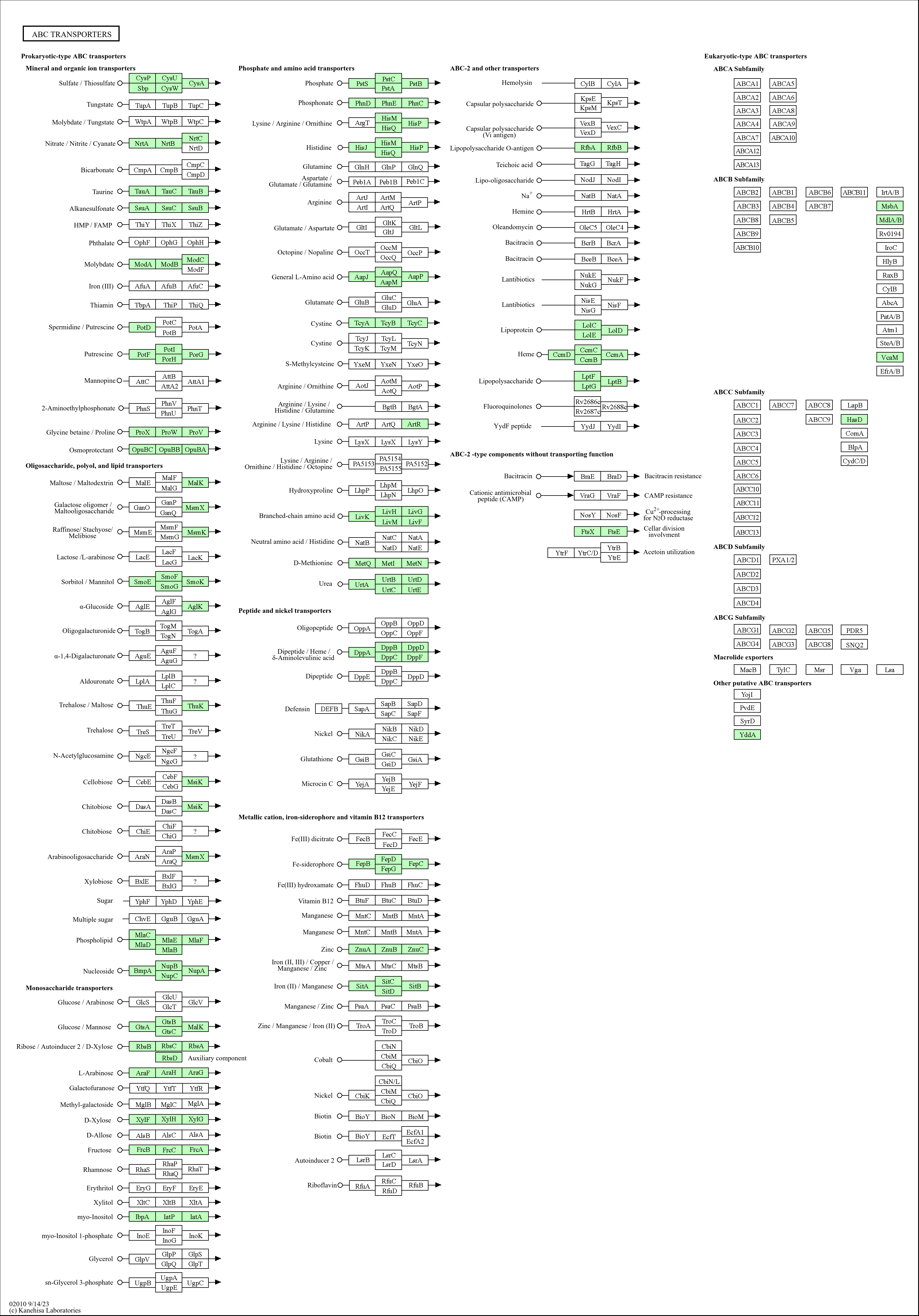


Figure S2. ABC transporters related genes available in the genome of P. loganensis sp. nov.


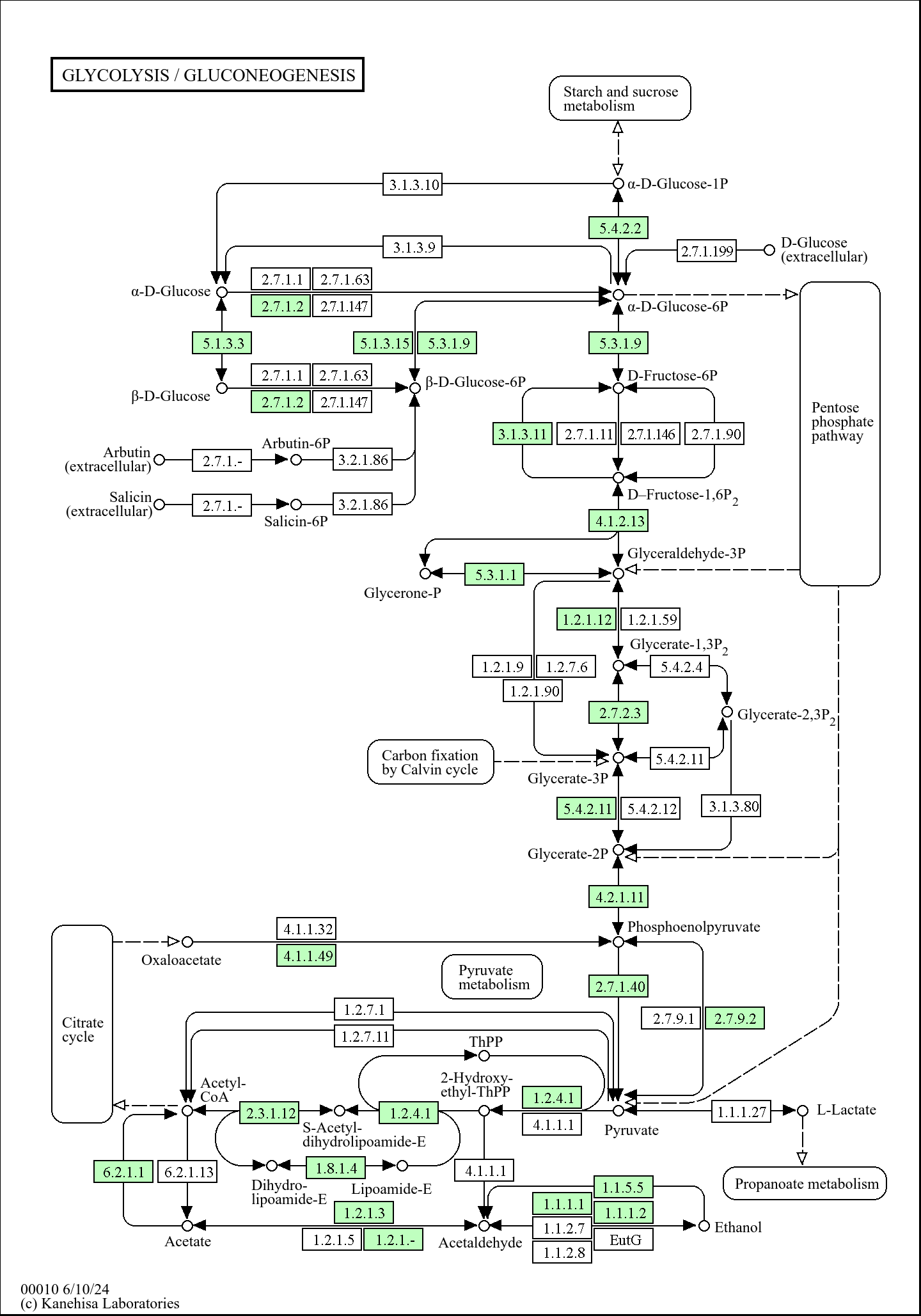


Figure S3. Genes involving in the glycolysis and gluconeogenesis in the genome of P. loganensis sp. nov.


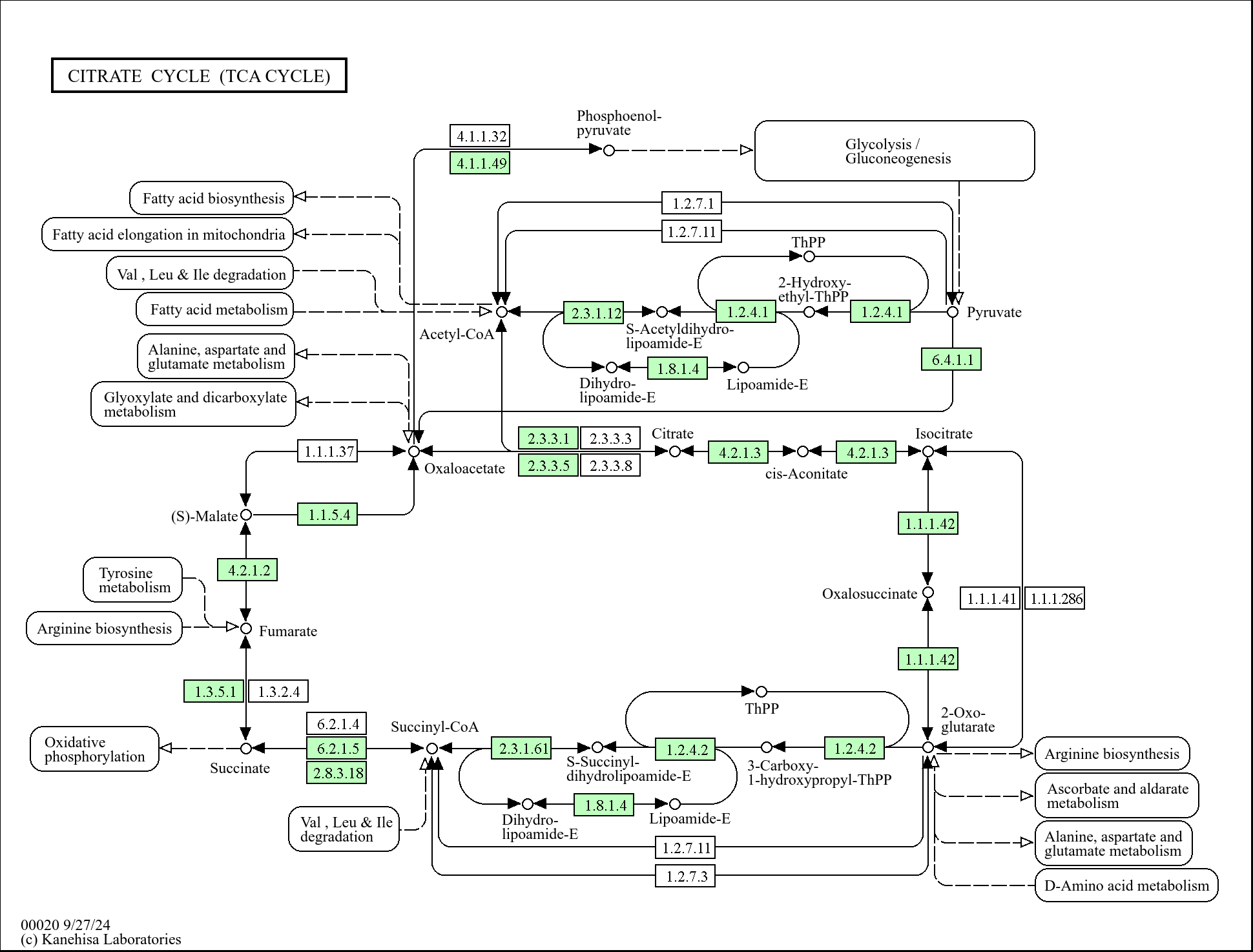


Figure S4. Genes involving in the Krebs cycle and methylcitrate cycle in the genome of P. loganensis sp. nov.


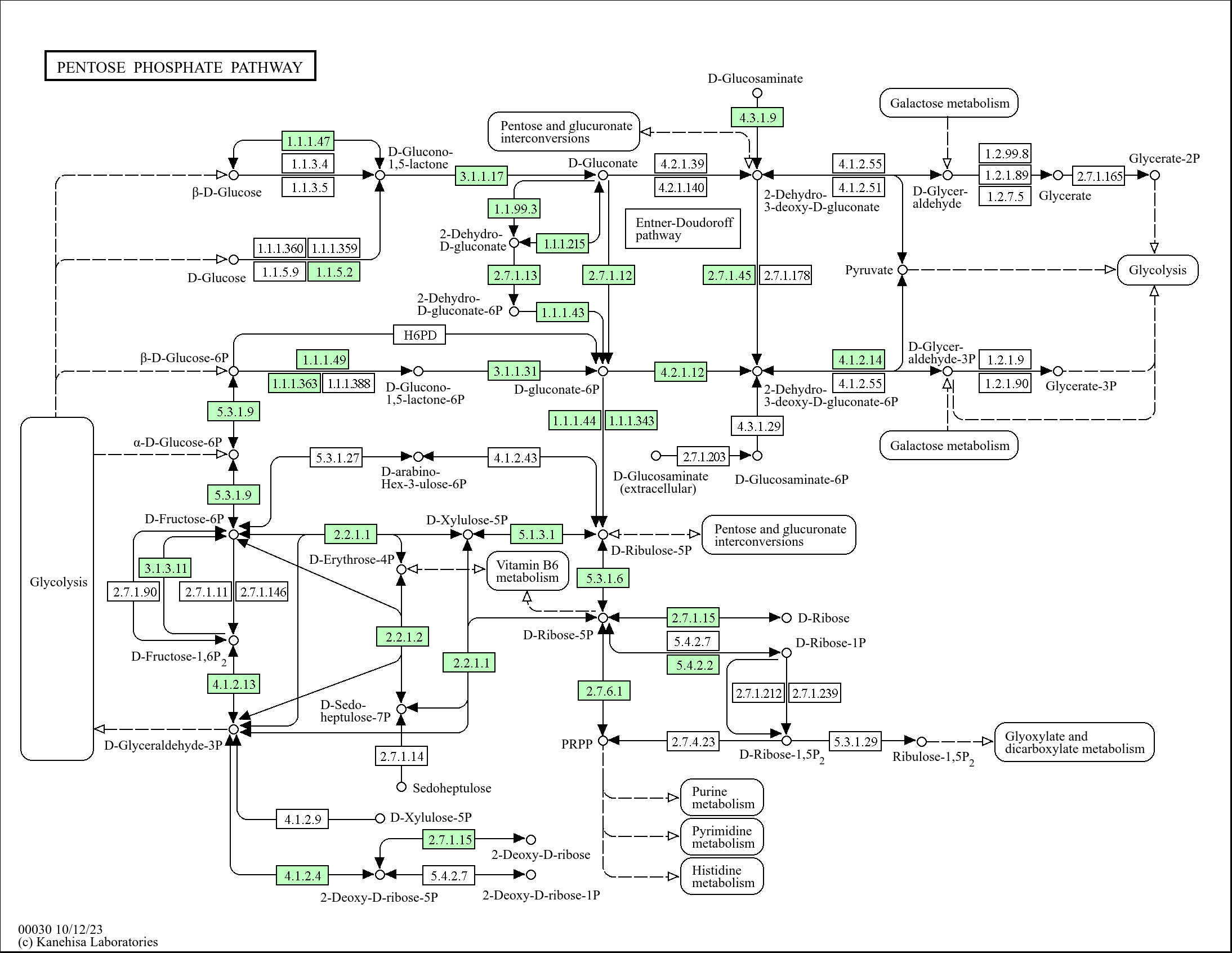


Figure S5. Genes involving in pentose phosphate and Entner-Doudoroff pathways in the genome of P. loganensis sp. nov.


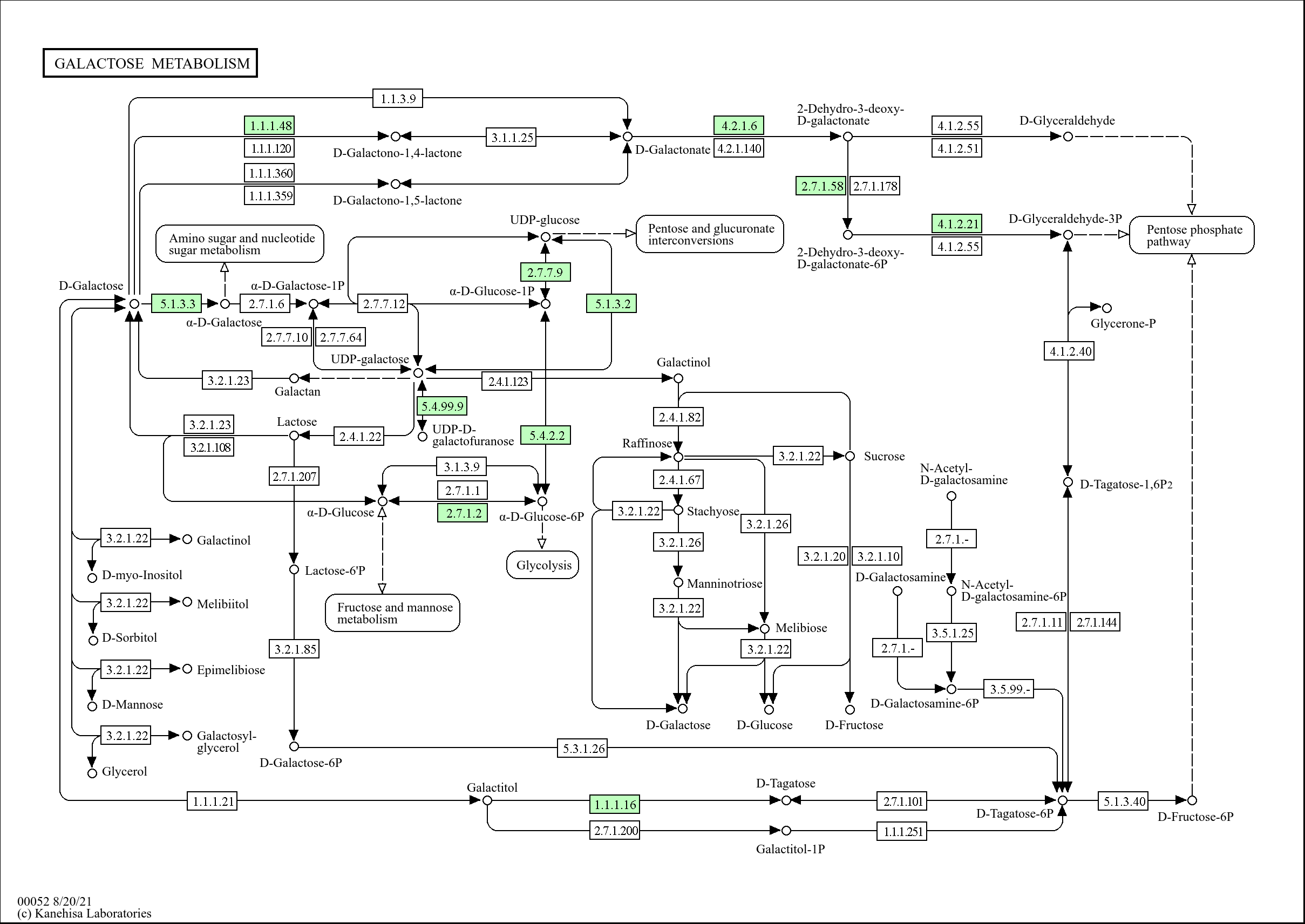


Figure S6. Genes involving in De Ley Doudoroff pathway for D-galactonate degradation in the genome of P. loganensis sp. nov.


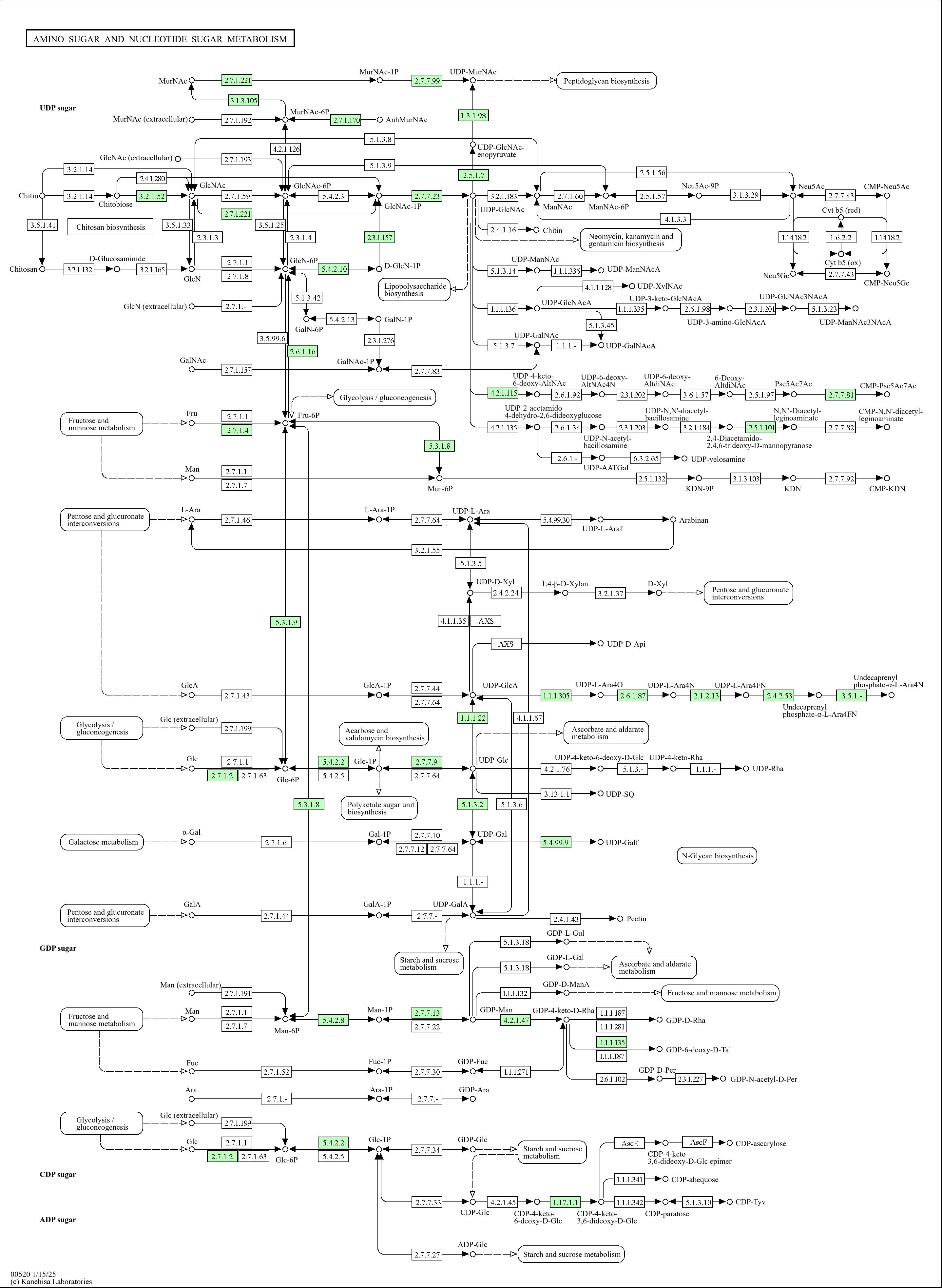


Figure S7. Genes involving in the biosynthesis of UDP-glucose, UDP-N-acetyl-D-glucosamine, and undecaprenylphosphate alpha-L-Ara4N in the genome of P. loganensis sp. nov.


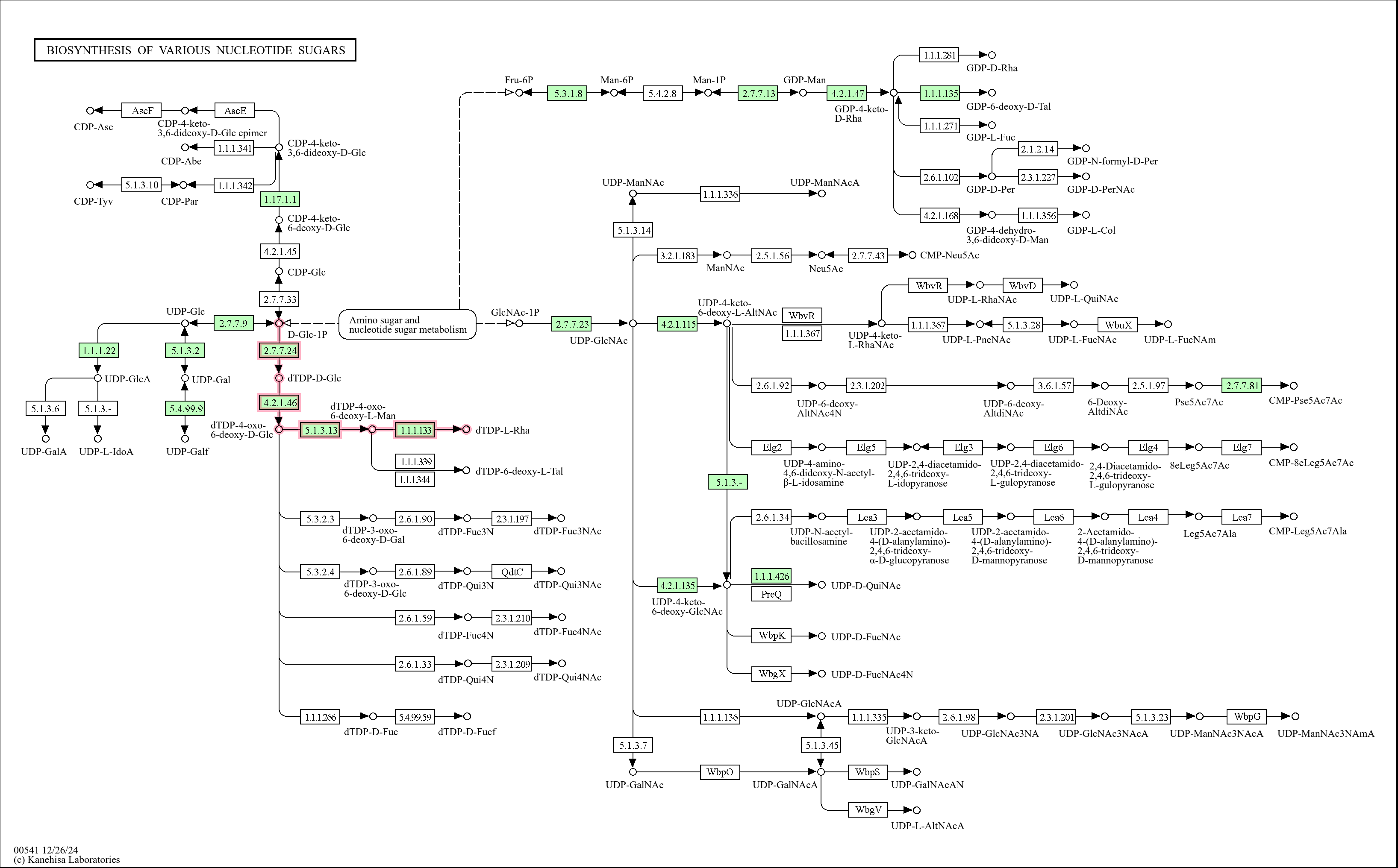


Figure S8. Genes involving in the biosynthesis of dTDP-L-rhamnose in the genome of P. loganensis sp. nov.


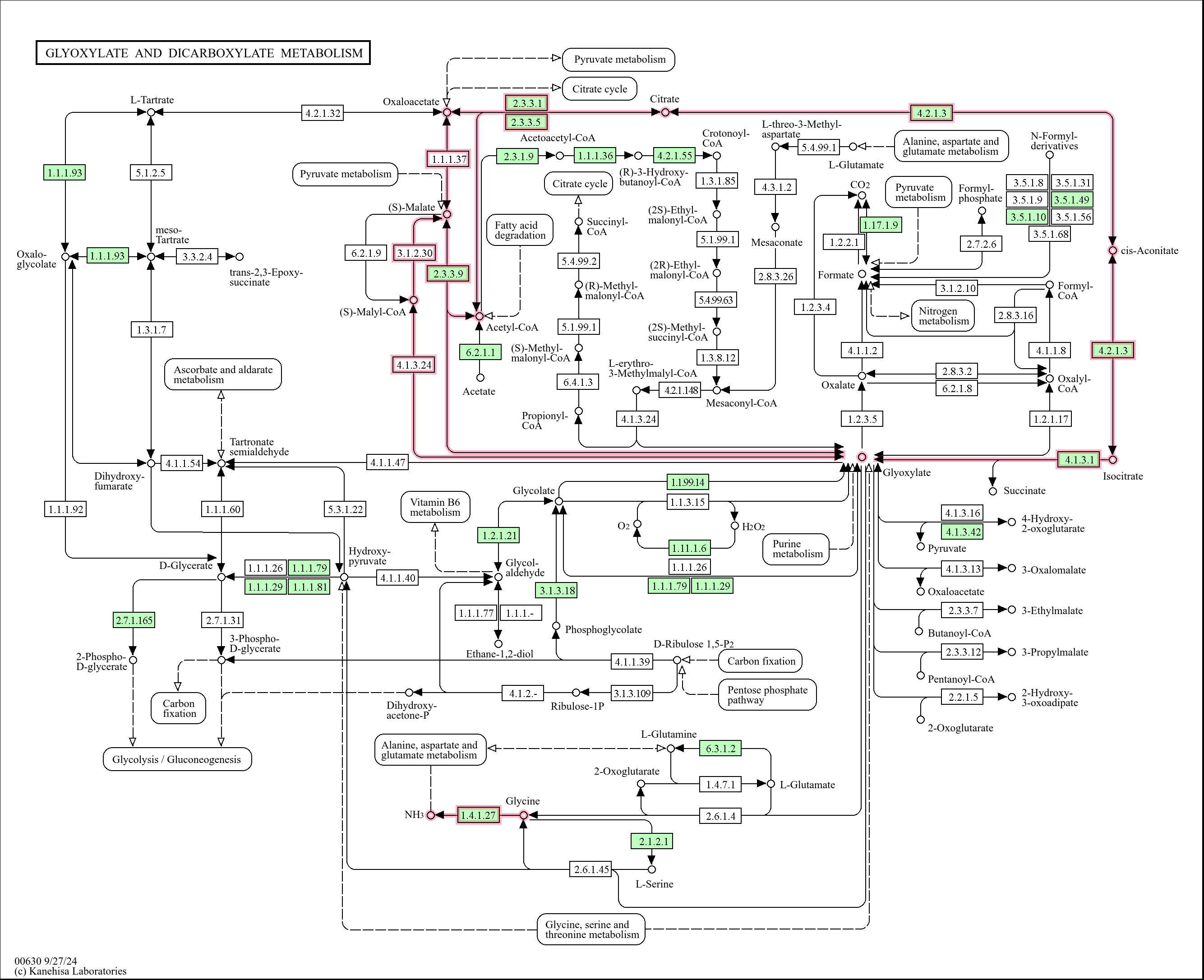


Figure S9. Genes involving in the glycine cleavage system and glyoxylate metabolism in the genome of P. loganensis sp. nov.


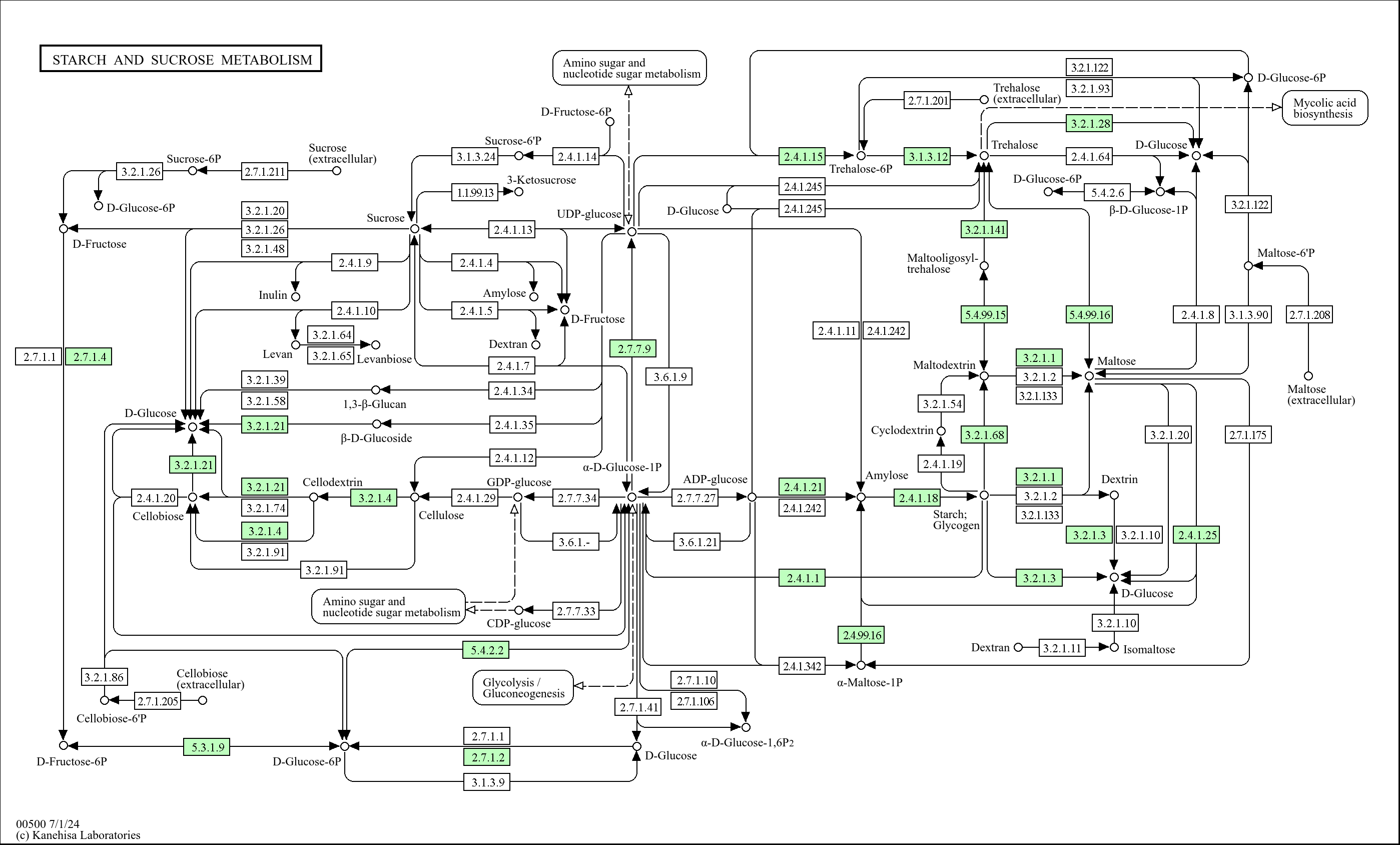


Figure S10. Genes involving in the starch and sucrose metabolism metabolism in the genome of P. loganensis sp. nov.


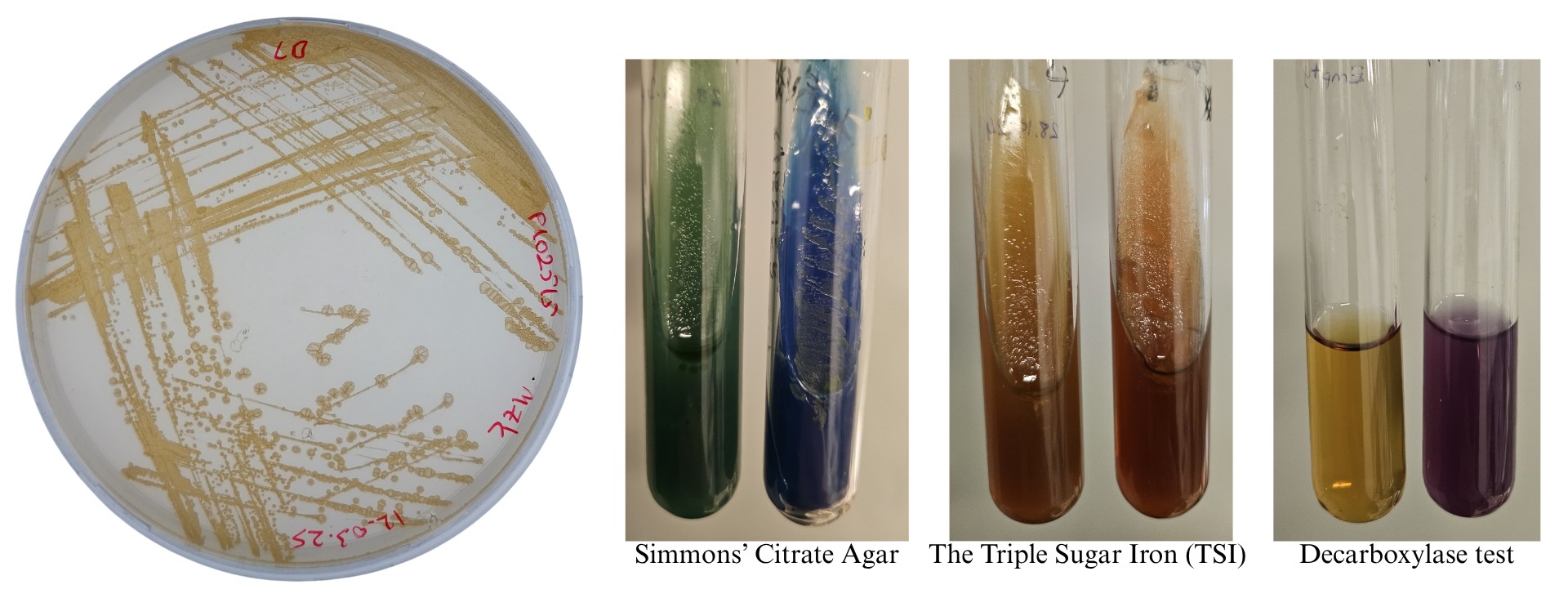


Figure S11. The images of a streak-plate with P. loganensis sp. nov. and of biochemical tests.


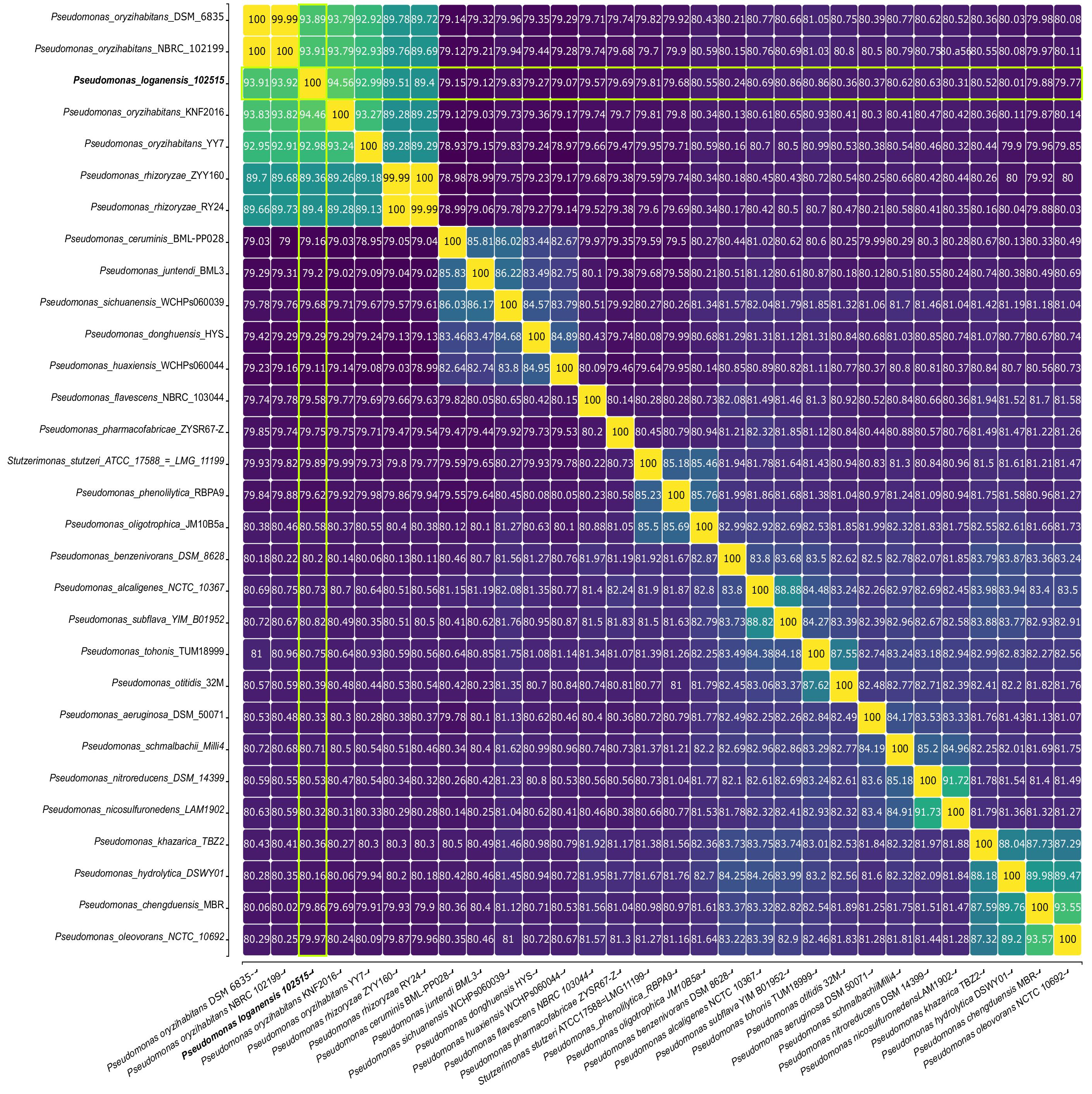


Figure S12. The heatmap illustrates the average nucleotide identity (ANI) phylogram of P. loganensis sp. nov. and other Pseudomonas members that were detected pursuant to aforecited neighbor joining trees. The heatmap was generated according to the FastANI algorithm. Based on FastANI values, P. loganensis sp. nov. showed a singleton feature.


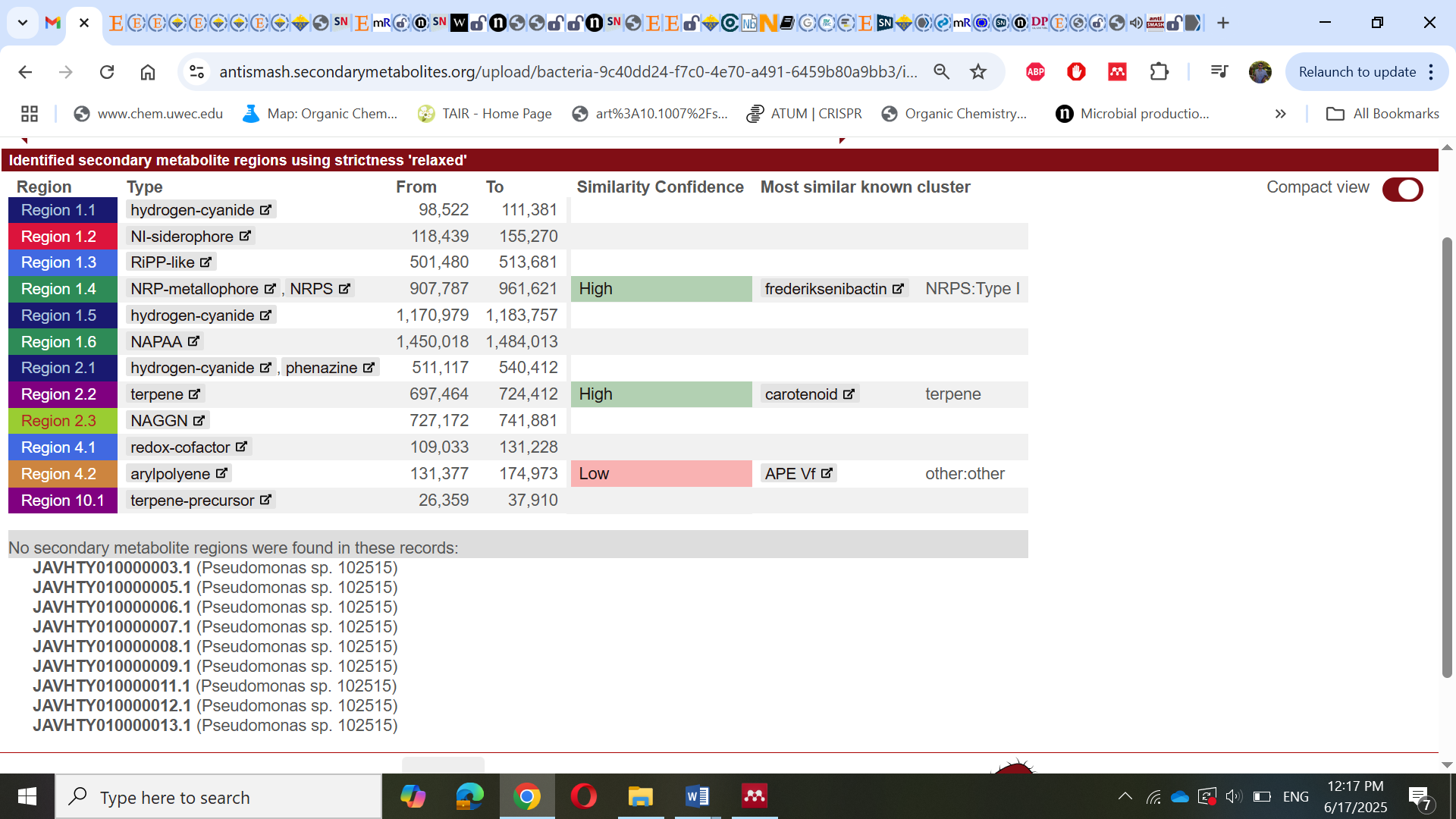


Figure S13. Overview of predicted biosynthetic gene clusters from the genome of P. loganensis sp. nov.


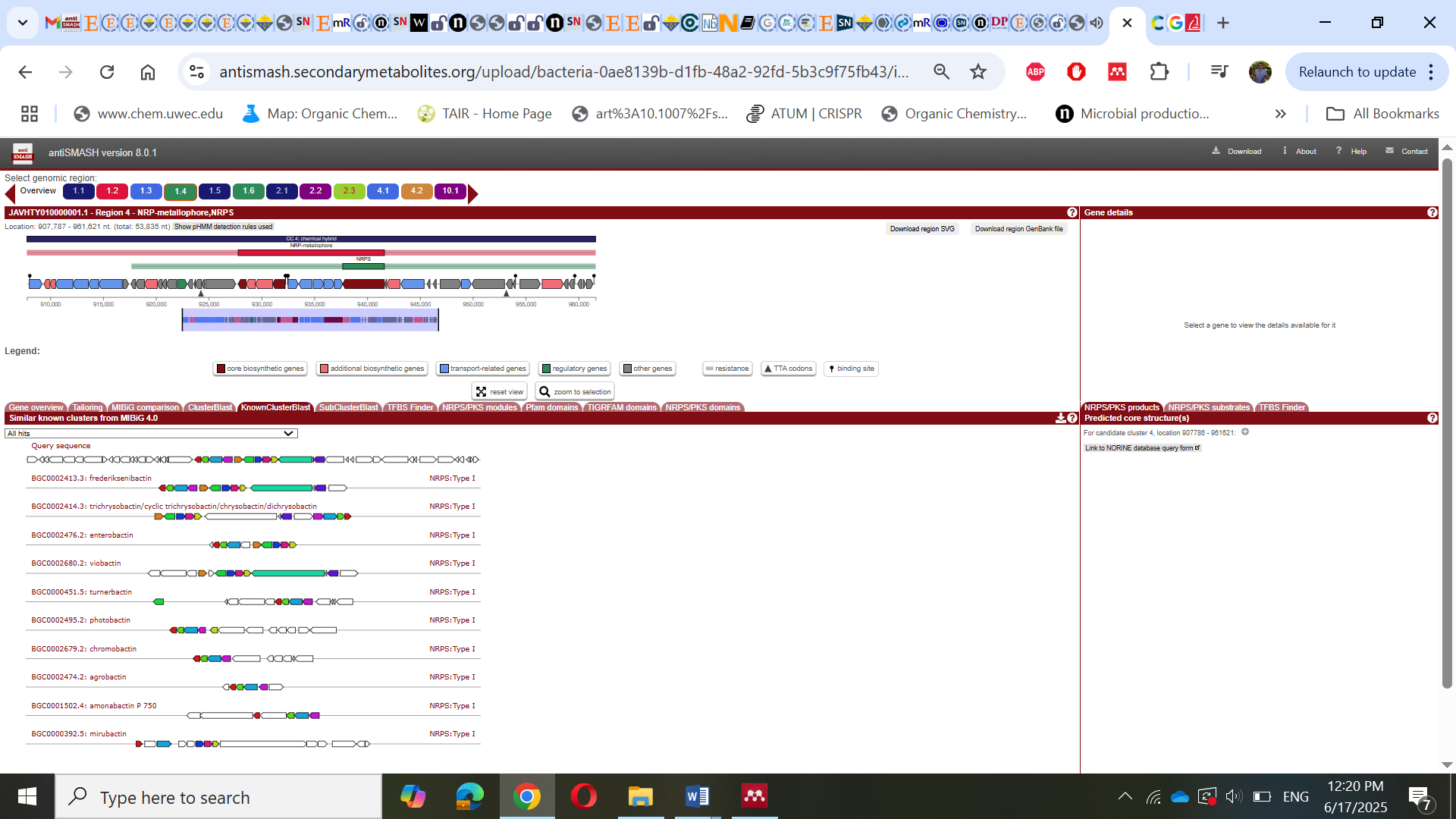


Figure S14. The details of siderophore biosynthetic gene cluster in the genome of P. loganensis sp. nov. and its comparison with known clusters.


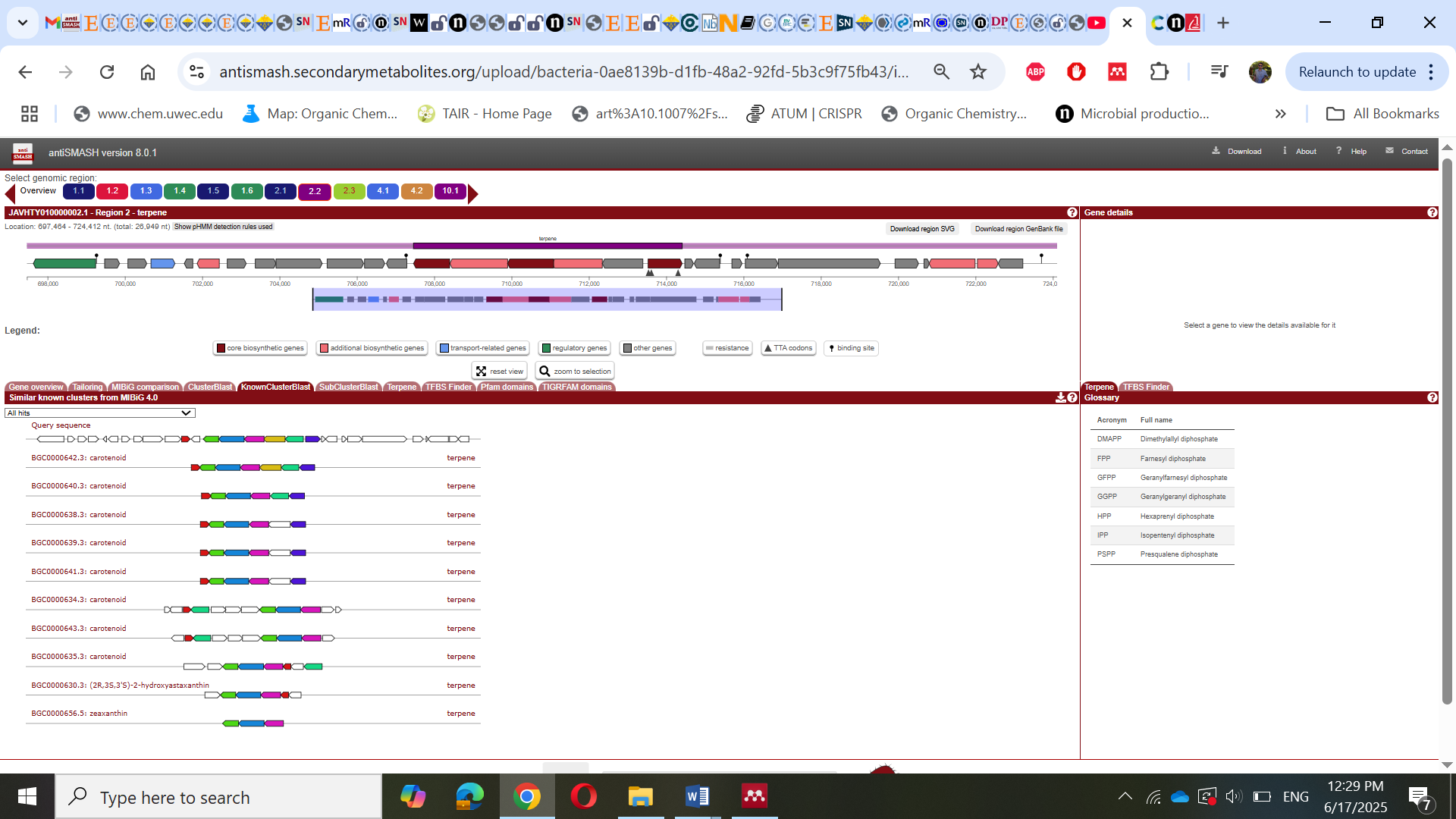


Figure S15. The details of carotenoid biosynthetic gene cluster in the genome of P. loganensis sp. nov. and its comparison with known clusters.
